# How being perceived to be an artist boosts feelings of attraction in others

**DOI:** 10.1038/s41598-023-45952-0

**Published:** 2023-10-31

**Authors:** Eugen Wassiliwizky, Paul Wontorra, Fredrik Ullén

**Affiliations:** 1 Department of Cognitive Neuropsychology, Max Planck Institute for Empirical Aesthetics, Grueneburgweg 14, 60322 Frankfurt am Main, Germany; 2 Goethe University Frankfurt, Frankfurt am Main, Germany

**Keywords:** Evolution, Psychology

## Abstract

Music production is a universal phenomenon reaching far back into our past. Given its ubiquity, evolution theorists have postulated adaptive functions for music, such as strengthening in-group cohesion, intimidating enemies, or promoting child bonding. Here, we focus on a longstanding Darwinian hypothesis, suggesting that music production evolved as a vehicle to display an individual’s biological fitness in courtship competition, thus rendering musicality a sexually selected trait. We also extend this idea to visual artists. In our design, we employed different versions of naturalistic portraits that manipulated the presence or absence of visual cues suggesting that the person was an artist or a non-artist (e.g., farmer, teacher, physician). Participants rated each portrayed person’s appeal on multiple scales, including attractiveness, interestingness, sympathy, and trustworthiness. Difference scores between portrait versions revealed the impact of the artistic/non-artistic visual cues. We thus tested Darwin’s hypothesis on both a within-subject and within-stimulus level. In addition to this implicit approach, we collected explicit ratings on the appeal of artists versus non-artists. The results demonstrate divergent findings for both types of data, with only the explicit statements corroborating Darwin’s hypothesis. We discuss this divergence in detail, along with the particular role of interestingness revealed by the implicit data.

## Introduction

### Why do humans engage with the arts?

Human engagement with the arts is present all around the globe and reaches far back into the prehistory of our species. Archeological findings date back to more than forty thousand years when it comes to musical instruments^1^ and paintings^2^. These artifacts show a stunning level of refinement and mastery, implying that the origins of music production and visual art must go back even way further into our past. This also holds true for art forms that do not depend on material objects, such as poetry, singing, or dancing, whose exact age is likely never to be determined^3^.

The first object our ancestors began to aesthetically modify were their own bodies, as suggested by archeological records of personal ornaments and ochre, a form of body paint, dating back at least 100.000 years^4,5^. These practices are believed to have primarily served the purposes of attracting sexual partners and demonstrating social status, aligning with our modern behaviors such as wearing make-up, jewelry, brands, and insignia. A longstanding hypothesis in evolutionary psychology extends this logic to the production of music. The considerable efforts invested into acquiring a skill as time consuming and strenuous as music production suggests that musicality must have held adaptive value for individuals in prehistoric societies, particularly given the rough living conditions and obligations of that era.

In his seminal work *The Descent of Man*^6^, Darwin proposes that human music production has its origins in sexual selection, specifically in courtship displays as they are used by many other species in order to signal genetic fitness and to impress the potential sexual partner. In Darwin’s own words: “musical notes and rhythm were first acquired by the male or female progenitors of mankind for the sake of charming the opposite sex” [6, p.336]. Typical examples in the non-human realm include birds, apes, and whales, especially male representatives of the respective species^7–11^. For humans, Darwin’s hypothesis is standing to reason given the emotional power and impact music can exert on listeners^12–14^, along with the visually displayed mechanical skills involved in playing an instrument^15^. Together, this multisensory stimulus signals extraordinary motoric, cognitive, affective, and communicative capabilities of an individual, which might have remained hidden otherwise (in contrast to facial attractiveness or physical fitness). The general tenet of Darwin’s idea was taken up and extended by a number of later theoreticians, particularly by Miller in the late 20th century^16–18^. It even came to prominence in the awareness of laypeople, as instructions on dating platforms such as Tinder imply, which (somewhat humorously) refer to using a profile image containing a musical instrument to increase one’s sex appeal (see Supplementary Fig. S1).

Despite its popularity and face validity, only a few studies have empirically substantiated Darwin’s idea. One online study demonstrated that friendship invitations from a Facebook profile featuring a man holding a guitar received more positive feedback from females (and higher acceptance rates) compared to the same profile without the guitar in the picture^19^. These results are in line with Darwin’s sexual selection hypothesis. However, while innovative in its design, the study suffers from substantial shortcomings, as it relies on only one stimulus and employs a between-subjects design, meaning that each female participant saw either the stimulus with or without the musical instrument.

A recent study used cross-modal priming to test Darwin’s hypothesis. Participants were presented with short piano excerpts before viewing facial pictures, thereby establishing an associative link between the auditory cue and the visual target^20^. The cues systematically varied in terms of arousal and complexity. The results partially supported the sexual selection hypothesis, as the cues did increase the perceived attractiveness and dating desirability for both sexes. However, this overall effect was not moderated as expected by arousal and musical complexity.

Another study^21^ utilized verbal descriptions of fictitious profiles, systematically manipulating whether the described person was musically active (as a lay musician, publically performing lay musician, professional musician) or not. The authors asked participants to rate the imagined attractiveness of the person described in the profile, as well as the motivation to engage with this person (i.e., meet, date, have a one-night-stand, have a long-term relationship). The results did not comply with Darwin’s hypothesis; profiles containing verbal cues about music production did not increase their attractiveness. Instead, the results were more in line with the similarity-attracts hypothesis^22^, where musicians preferred musicians and non-musicians preferred non-musicians. One limitation of this study is once again the use of a between-subjects design and a small number of ratings per condition. Another drawback is the task itself. While attractiveness is largely based on visual processing, participants here had to provide attractiveness ratings based on textual material, which did not include any cues about the person’s visual appearance. Although not presenting a photograph and relying solely on imagination could be considered a potential strength of this design, it might also have made it more difficult for the effect to emerge.

Similarly, a study from the field of behavioral genetics found little support for Darwin’s idea^23^. Analyses of twin modelling in a large genetically informative sample revealed no significant relation between musical ability and mating success. However, it is worth noting (as discussed by the authors in the limitations section of their article) that most of the measures used in these analyses were not originally designed to test the sexual selection hypothesis. Instead, the authors had to use available variables from the sample and match them as closely as possible with the concepts relevant for Darwin’s prediction.

In sum, to date, only a handful of studies have dedicated their attention to testing the sexual selection hypothesis, presenting evidence both in favor and against Darwin’s idea. Additionally, most of these studies suffer from various limitations. Given this state of affairs, it is difficult to conclusively determine the validity of the sexual selection hypothesis. Thus, claims refuting the hypothesis based on missing evidence^24,25^ could turn out to be premature, as the absence of evidence is not evidence of absence. Much more data are needed to confirm or refute Darwin’s idea. The current study aims to contribute a piece of evidence to the body of research and overcome some of the limitations of previous work.

It should be noted that Darwin’s line of thinking is not the only evolutionary approach to human musicality. Alternative accounts, for instance, emphasize the power of music to promote mother–child bonding, as in Ellen Dissanayake’s work, or facilitate social bonds in general. In contrast to focusing on in-group cohesion, some theorists bring into focus that music production can also be used for out-group exclusion, with martial music accompanying, for instance, war dances being typical examples for this function. Yet other theorists, most notably Steven Pinker, argue that musicality is a useless by-product of the evolution of the human central nervous system. A comprehensive overview and thorough discussion of alternative theoretical accounts on the evolution of musicality are presented in^24,25^. Some of these theories are not mutually exclusive with Darwin’s reasoning. In this study, however, we will exclusively focus on Darwin’s suggestion.

### Current study

The basic idea of the present study was to provide participants with two versions of a person’s photograph, similar to Tifferet et al., but in a within-subject setting. For this purpose, participants were invited to the lab twice, with a one-month gap between the sessions. One version of the photograph included cues related to the musical status of the depicted person, while the other version had no such cues. Consistent with with the sexual selection hypothesis, we anticipated that cues rendering a person a musician would boost their perceived attractiveness (Hypothesis 1a).

While our primary focus was on music production, we also aimed to investigate the sexual selection hypothesis in relation to visual artists by incorporating portraits of painters and photographers (Hypothesis 1b). In general, we see no reason why other art forms could not have a similar enhancing effect on a person’s perceived attractiveness as music is believed to have, especially given that alongside musicality, aesthetic expression in the visual domain is among the oldest forms of our species. In fact, in his own writings, Darwin applied the same logic to other art forms, notably dancing and poetic language^6,26^.

One could argue that adding any cues to a person’s portrait, which reveal their specific professional interests, might enhance their perceived attractiveness, regardless of their artistic status. For instance, providing cues that indicate a person is a teacher or a doctor could have a similar boosting effect on their perceived attractiveness. In his own writing, Darwin explicitly juxtaposes the human capacity for music production with more practical skills, which he refers to as “daily habits of life” [6, p.333]), that directly support an individual’s survival and wellbeing, as well as that of society on a large scale. Building on this notion, we aimed to compare musicians and visual artists with individuals specializing in non-artistic fields as a control group. We expected that, overall, the enhancing effect would be specific, or at least particularly pronounced, for musicians and visual artists, while being weaker or even absent for individuals with other specializations (Hypothesis 2). Translating the practical “habits of life” into modern terms, we included a wide range of portraits that provided cues about a person’s non-artistic vocational interests as part of the control group (for further details, please see the Stimuli section).

In accordance with Darwin’s line of thinking, we also anticipated that the enhancing effect would be strongest in cross-gender direction, while being weaker or even absent when participant viewed individuals of their own gender (Hypothesis 3a). It is worth noting that, in contrast to later authors^27^, Darwin himself did not propose a sexual dimorphism in musicality, where males would have superior abilities compared to females, as is often observed in the animal kingdom, such as in songbirds. In fact, Darwin even assumed the opposite: “Women are generally thought to possess sweeter voices than men, and as far as this serves as any guide, we may infer that they first acquired musical powers in order to attract the other sex.” [6, p.337].

Finally, to consider the possibility of ceiling effects, we anticipated that the enhancing effect would be most pronounced for faces with average or lower attractiveness (Hypothesis 3b). In other words, for individuals with highly attractive faces, adding attributes related to their artistic status would not have a measurable incremental effect. In a related vein, recent studies on mate choice have demonstrated that lower facial attractiveness can be compensated for by an individual’s aesthetic creativity (for language, see^28^; for music, see^29^).

In addition to attractiveness, we also collected ratings for sympathy, interestingness, and trustworthiness, as well as the participants’ motivation to meet the depicted person. We did this to acquire a more detailed understanding of which related appealing qualities might be influenced by the boosting mechanism. While trustworthiness and personal sympathy are well-established factors in mate choice^22,30^, we were also intrigued by how people perceive a person’s level of interestingness, a quality often considered important in research on art and aesthetics, including painted portraits, but rarely explored in experimental studies on mate choice. We included interestingness into our current study, as we do not draw a strict distinction between mundane versus art-related aesthetic judgment^31^.

Finally, we also obtained an implicit measure of how much time participants spent viewing each portrait before switching the screen (via a button press) to submit their ratings (for more details, please refer to the Procedure section). To summarize, we formulated five a priori hypotheses and preregistered them on the Open Science Framework (https://osf.io/29ynx):

#### H1a

Attributes rendering a person a musician boost their perceived attractiveness.

#### H1b

Attributes rendering a person a visual artist boost their perceived attractiveness.

#### H2

The boosting effect is specific for artists and weaker or absent for other professions.

#### H3a

The boosting effect is strongest in cross-gender direction.

#### H3b

The boosting effect is strongest for faces with average or low attractiveness.

Technically speaking, H2, H3a, H3b represent moderations of H1a and H1b. Analogous hypotheses apply to sympathy, interestingness, trustworthiness, the wish to meet, and the self-paced viewing time of a portrait.

Please note that the design of this study captures the potential boosting effect implicitly, since participants were not informed that each person would appear twice during the testing, once with cues on artistic status or other specialization and once without. Even if participants recognized the face of a depicted person during the second session of the experiment, it would be virtually impossible for them to remember which ratings they provided for the other version of the person’s portrait in the first session. This is due to the one-month gap between the sessions and the substantial number of ratings (specifically, 950) that participants had to provide in each session. In addition to this implicit approach, we were also interested in whether participants explicitly considered artistic individuals to be more attractive (sympathetic, interesting, and trustworthy) than non-artistic people. We collected these explicit ratings at the very end of the second session (for further details, please refer to the Procedure section).

## Methods

### Stimuli

In the first step, we collected a large pool of almost a thousand high quality, half-length portraits from the internet, featuring musicians, visual artists, and various non-artistic vocational categories. We sourced these stimuli from actual images found on personal and commercial websites, the press, as well as stock photography. The half-length (or torso) portraits depicted individuals in everyday settings, providing cues about their artistic status or their other specialization. For instance, in the case of musicians, the portraits showed individuals sitting at a piano, harp, or drum set, or holding musical instruments such as a flute, an e-guitar, or a microphone (for singers). We made sure to represent a wide spectrum of instruments and musical styles, including classical, rock, pop, and electronic music (Table 1).

**Table 1:** Number of half-length portraits for subgroups and the three status groups.

Group	Gender		Total
	f	m	
Musicians			69
Guitarists	6	6	
Key-instrumentalists	4	4	
Harp-players	3	3	
String-players	6	6	
Brass-instrumentalists	6	6	
DJs	3	3	
Percussionists	3	3	
Singers	3	4	
Visual artists			16
Painters	5	5	
Camera operators	3	3	
Non-artists (control)			105
Farmers	3	3	
Military-personnel	3	3	
Craftsmen	7	7	
First-responders	3	3	
Florists	3	3	
IT-technicians	3	3	
Journalists	3	2	
Mechanists	4	4	
Policemen	4	4	
Physicians	3	3	
Officials	3	3	
Athletes	8	10	
Teachers	2	2	
Waiters	2	2	

In the second step, we systematically reduced the number of stimuli. Particular attention was given to balancing genders within and across all categories and subcategories, as well as their level of attractiveness. Our goal was to provide a diverse range of physical facial attractiveness while avoiding any systematic differences between artists and non-artists. This pre-selection process was based on judgments from a select group of highly experienced raters within our lab. However, the data collected from the actual sample validate our pre-selection, as discussed later in the manuscript (please also refer to Fig. S2).

**Figure 1:**
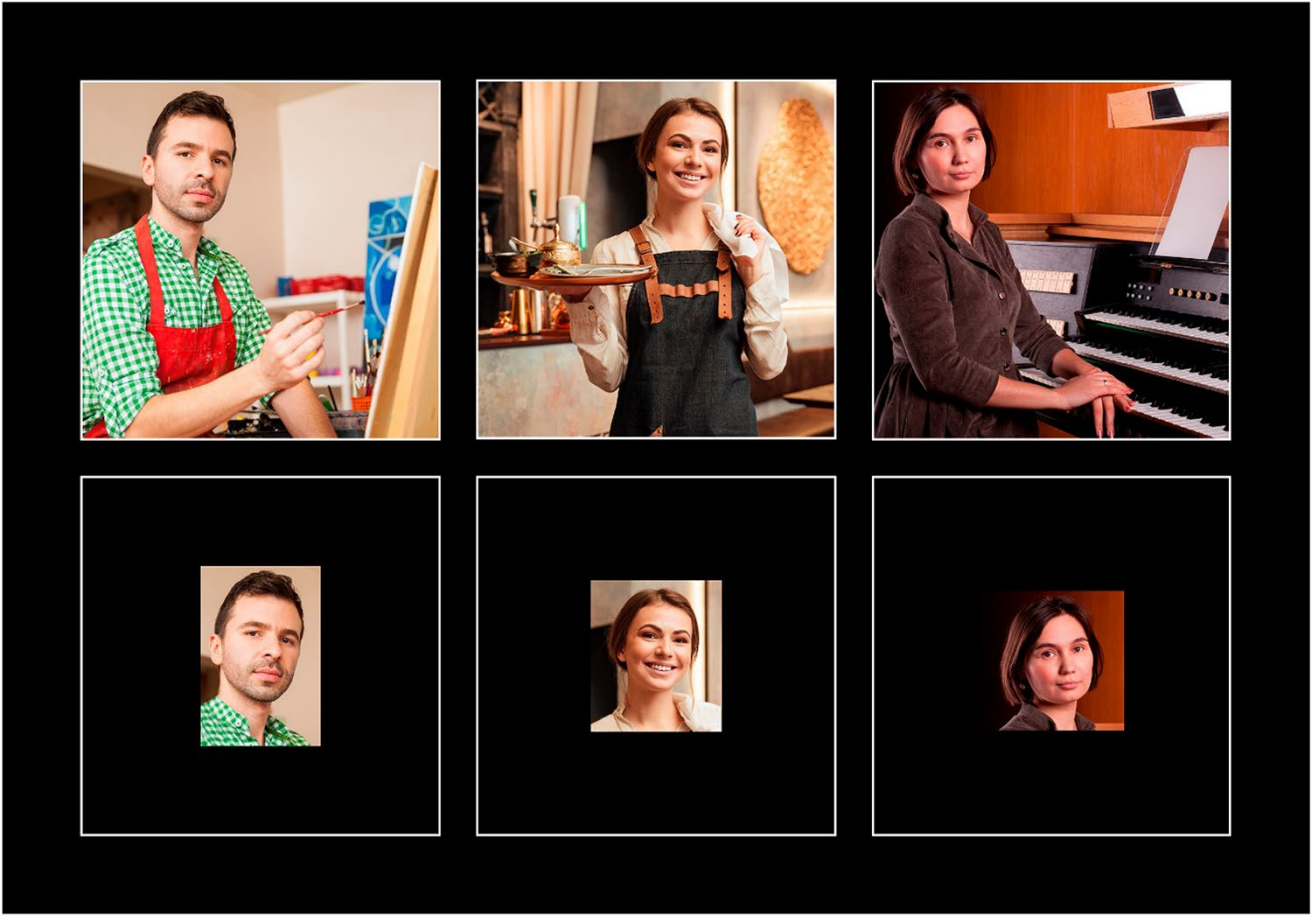
Stimulus examples. The upper row displays half-length portraits featuring a painter (left), a waitress (middle), and a musician (right), in this case, an organ player. The lower row showcases facial portraits, which are cropped versions of the same images, where all cues related to musical/artistic status, or other specialization, have been removed. (These images represent purchased stock footage material, licensed for publication in an open access journal.)

Finally, we excluded all portraits that were not suitable for our intended manipulation. This involved cropping the half-length portraits to facial portraits and, if necessary, retouching any remaining cues that might hint at a person’s artistic status or vocational interests. In the cropped version, all faces where centered in the image and surrounded by a black frame. The size of the face remained identical across the two versions of portraits. The final stimulus set consisted of 380 portraits, comprising 190 contextualized half-length portraits and 190 de-contextualized facial portraits derived from the former (for examples, see Fig. 1).

### Participants

Eighty German-speaking participants (42 women and 38 men, with a mean age of 33.06, *SD* = 12.34, ranging from 20 to 74) were invited to the lab for a study titled ‘Faces in Everyday Life,’ as stated in the announcement. Participants were recruited through advertising on the university campus and from our institute’s internal participant database, which includes a substantial proportion of individuals who are not students. This contributed to the aforementioned mean age of 33 years. All participants provided informed consent and were compensated with either 40 Euros or course credit after the second session of the experiment. All participants had normal or corrected-to-normal vision and none was excluded from the analysis. The manuscript does not contain any information that could lead to the identification of a study participant.

### Task and procedure

To ensure a private atmosphere, participants completed the experiment while sitting alone in a testing room. At the outset, they were instructed that in this rating study, they would be presented with photographs of people portrayed in everyday-life situations. Their task would be to provide subjective judgments about the depicted individuals using five different scales. We provided minimal context, not mentioning that two different portrait layouts would be presented, that characters would appear more than once during the study, or that the half-length portraits would include clues regarding the artistic vs. non-artistic interests of the depicted characters.

Presentation®22.1 (Neurobehavioral Systems, San Francisco, USA), including a custom-made open-source user interface^32^, was used to display all instructions, stimuli, and scales on a 24″ high-definition screen with a resolution of 1920 by 1080 pixels. The first session commenced with general instructions and a test trial, followed by an opportunity for participants to ask questions via an intercom system. Each portrait was presented in full-screen and remained onscreen for a self-paced duration. Participants could click a button to reveal the scales in order to submit the ratings. The duration of self-paced stimulus exploration before rating was captured and used later as an implicit measure (Viewing Time).

**Figure 2:**
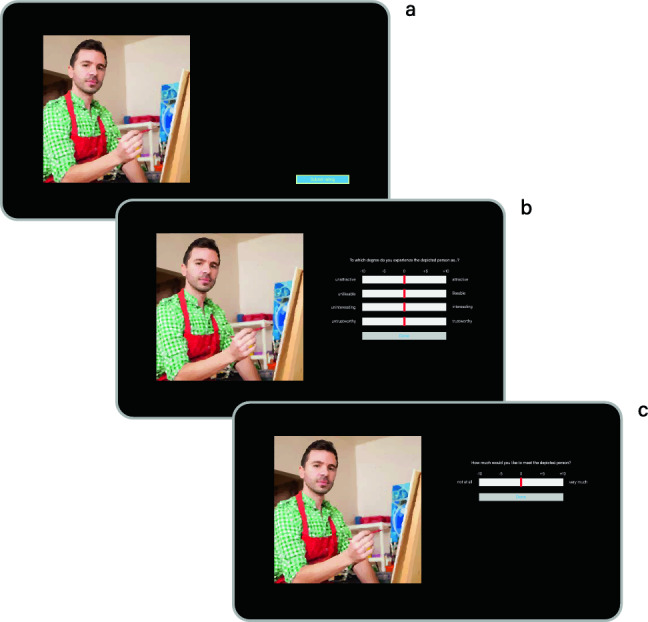
Schematic diagram of a trial. (**A**) The first screen displayed a portrait on the left half and a ‘Submit Rating’ button in the lower right corner. The self-paced exploration time (i.e., the time gap between the appearance of the portrait and the participant’s clicking of the button) was recorded and used as an implicit measure for attentional binding and thus charming qualities of the depicted person. (**B**) Upon clicking the button, bipolar scales, ranging between  − 10 and  + 10, and a red cursor initially placed at the midpoint of each scale (i.e., at position 0), appeared on the right half of the screen. After completing the ratings, participants had to click the ‘Done’ button below the scales. (**C**) The wish to meet the depicted person was measured on a separate screen.

The ratings for the target concepts of Attractiveness, Interestingness, Sympathy, Trustworthiness, and Wish-to-Meet were assessed using a movable cursor on bipolar scales ranging from  − 10 to + 10. The poles of the scales were verbally anchored with adjective antonyms: ‘unattractive, attractive’, ‘unlikable, likeable’, ‘uninteresting, interesting’, and ‘untrustworthy, trustworthy’. The question above the scales read: ‘To what degree do you experience the depicted person as…?’. The participants’ task was to position the cursor at the appropriate point, and we recorded their responses with a finer resolution than presented to them, specifically within a range between  − 50 and + 50. The motivation to meet the depicted person was measured using a similar scale, but on a separate screen (see Fig. 2). The question here read: ‘How much would you like to meet the depicted person?’ with verbal anchors ‘not at all’ and ‘very much’.

Please note that, although we are aware that the mechanism of sexual selection, as described by Darwin, ultimately focuses on sexual contact, we refrained from directly asking how much participants would like to have sex with the depicted person. This wording was perceived as inappropriate in pilot sessions, particularly by participants above 30 years of age. However, we are convinced that asking about ‘feelings of attractiveness’ effectively captures the judgment we intended to measure, as in German, the semantics of ‘attraktiv’ carry a strong connotation of romantic attraction. Since our participants were German native speakers, we can rule out the possibility that they misinterpreted the target concept as Platonic interest in another person. Furthermore, informal conversations we had with the participants after the second session confirmed our assumption that they understood the ratings as we intended.

In each session, participants were presented with a total of 190 portraits. The order of the stimuli within each session was randomized for each participant. Additionally, we prepared two versions of the experiment to counterbalance whether a person’s torso or face was presented in the first session. Each session lasted approximately 60 min per participant, with self-paced breaks available during the session.

The second session occurred at least three weeks after the first one, with an average time gap between sessions of 27.6 days, and it followed a similar structure. However, at the conclusion of the second session, we collected additional data consisting of:

- Sociodemographic information, which encompassed participants’ own musical and artistic status
- Participants’ self-perceived own attractiveness, sympathy, interestingness, and trustworthiness
- Participants’ personal preferences regarding the professions they encountered in the study
- A section for participants to report any observations or comments they wished to communicate

Data from (a) to (c) were collected for potential use as covariates in subsequent analyses, while (d) aimed to determine whether participants noticed that each face appeared twice during the testing; eight participants, constituting 10% of the sample, reported to have noticed this. As a final step, we asked participants to explicitly compare artistic individuals with non-artistic individuals using 7-point bipolar Likert scales. These scales included ‘unattractive, attractive’, ‘unlikeable, likeable’, ‘uninteresting, interesting’, ‘untrustworthy, trustworthy’, ‘uneducated, educated’, ‘incompetent, competent’, ‘distanced, empathic’, ‘unsociable, sociable’, ‘boring, exciting’, ‘unintelligent, intelligent’, and ‘unreasonable, reasonable’. The statement preceding the scales read: ‘Artistic people (i.e., musicians, painters, photographers, singers, actors, etc.) are compared to non-artistic people more…’ The verbal anchors along the scales were ‘very much’ (positioned at 3), ‘considerably’ (positioned at 2), ‘somewhat’ (positioned at 1), and ‘similar’ (i.e., not different, positioned at 0 at the center of the scale).

The study was conducted in accordance with the Declaration of Helsinki. All methods and procedures were approved by the Ethics Committee of the Max Planck Society (approval 2017_12).

### Analysis

The study followed a 2-by-3 factorial design, with the portrait’s Layout (torso vs. facial) and the Status of the depicted person (musician vs. visual artist vs. non-artistic occupation) as factors manipulated within each participant. We also included the gender of both the participants and the depicted individuals (male vs. female) as moderating factors to test Hypothesis 3a. Similarly, we included the mean rating for each facial portrait (averaged across all 80 participants) as a moderating covariate to examine Hypothesis 3b (later referred to as mean facial attractiveness). No centering of predictors was performed since they resemble categorical factors, except for the portraits’ mean ratings, which were centered around the grand mean prior to fitting the models. Additionally, the time durations of portrait viewing before submitting the ratings, measured by Presentation in 10^−4^ s, were log-transformed to remove the skewness of the original data.

As preregistered, we employed linear multi-level modeling (LMM) to investigate our hypotheses since the data exhibit a nested structure, with portraits at Level 1 and participants at Level 2. All statistical analyses were conducted in R^33^ using the *lme4* package version 1.1–27^34^. Restricted maximum likelihood (REML) estimation method was applied to obtain the parameter estimates. P-values were obtained using the *lmerTest* package^35^. Following a recent review’s recommendation^36^, we also report the profile likelihood confidence intervals (PLCI) with 95% boundaries (in square brackets following each parameter). These intervals were computed using the *confint* function within the *lme4* package. Effect sizes are reported as *d* measures following Westfall et al.^37^’s recommendation, calculated as the estimate for fixed effect divided by the square root of the pooled variance of random effects.

All data and analysis scripts, including models and code necessary to replicate the figures and tables, are available on the Open Science Framework. To visualize the results, we computed estimated marginal means (EMMs), which include 95% confidence intervals as error bars^38^, using the *emmeans* package version *1.7.2*^39^. Post-processing of figures was carried out using Adobe Illustrator.

## Results

### Explicit comparison between artists and non-artists

**Figure 3:**
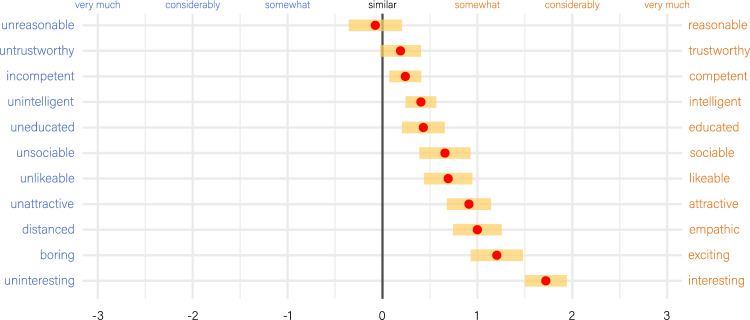
Explicit comparison of artists to non-artists. Red dots represent means across all 80 participants. Error bars represent 95% confidence intervals. Ratings for scales that do not include the 0-line are significantly different from zero in one-sample t-tests at *p* < *0.01*.

When participants were asked at the end of the study to explicitly compare artistic and non-artistic individuals, they favored the artists on almost all scales, except for Trustworthiness and Reason. Particularly notable was their positive judgment regarding Interestingness (Fig. 3).

### General observations

Before delving into the results of our main hypotheses, we would like to report on two general findings. First, the results of an LMM predicting viewing times based on the mean facial attractiveness indicate that more attractive faces were associated with longer self-paced viewing times for both layout formats (facials: *ß* = *0.001 [0.001, 0.002], t* = *2.657, p* = *0.009, d* = *0.002*; torsos: *ß* = *0.001 [0.001, 0.002], t* = *2.164, p* = *0.032, d* = *0.002*; for statistical details, refer to Supplementary Table S1). These delays in participant responses prior to rating submission suggest attentional engagement and the rater’s interest in the depicted person. This lends support to our use of viewing times as an implicit measure for the outer appeal of the depicted person.

**Figure 4:**
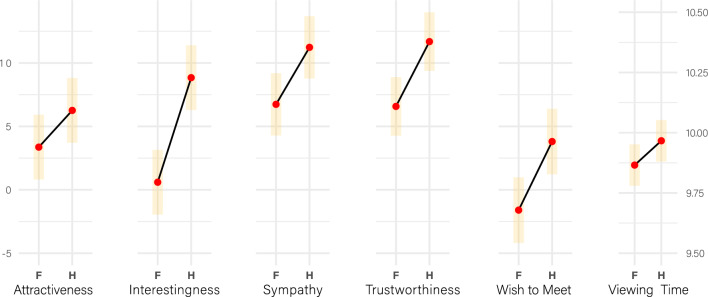
Contextual Enhancement Effect. The plots depict estimated marginal means, including their 95%-CI limits. In general, individuals were perceived more positively and captured viewers’ attention for a longer duration when presented in half-length portraits (**H**), which convey contextual information about the person’s vocational interests, as opposed to facial portraits (**F**). For statistical details, please refer to Supplementary Table S2. Note that Viewing Time has a different scaling than the rating scales.

Second, the results of LMMs, in which we predicted the entered ratings based on the layout format, indicate that half-length portraits generally received substantially higher scores on all scales (Fig. 4) and were viewed longer than facial portraits (for statistical details, refer to Supplementary Table S2). In itself, this is an interesting and, to our knowledge, previously unreported phenomenon, which we will term ‘contextual enhancement effect’. Our main multi-level analyses below elaborate on this general boosting mechanism in terms of its specificity (H2) for musicians (H1a) and visual artists (H1b), as well as the moderating role of gender (H3a) and the initial facial attractiveness of the depicted person (H3b). In statistical terms, specificity is reflected in the interaction between Layout and Status. Visually, specificity pertains to the degree of steepness from faces to torsos within each individual status group (musicians, visual artists, non-artists) for each dependent variable.

### Attractiveness

In order to examine the specificity of the contextual enhancement effect for musicians and visual artists compared to non-artists, we tested the following multi-level model (Model-A), in which we allowed intercepts to vary between participants and stimuli:

$${\text{Level}}\;1:\quad {\text{Rating}}_{{{\text{ij}}}} = { {\upbeta }}_{{0{\text{j}}}} + { {\upbeta }}_{{{\text{i}}0}} + { {\upbeta }}_{{1{\text{j}}}} {\text{Layout}}_{{{\text{ij}}}} \times { {\upbeta }}_{{2{\text{j}}}} {\text{Status}}_{{{\text{ij}}}} + { {\upvarepsilon }}_{{{\text{ij}}}}$$

$$\begin{aligned} {\text{Level}}\;2:\quad { {\upbeta }}_{{0{\text{j}}}} = & { {\upgamma }}_{{00}} + v_{{0{\text{j}}}} \\ { {\upbeta }}_{{{\text{i}}0}} = & { {\upgamma }}_{{00}} + v_{{{\text{i}}0}} \\ { {\upbeta }}_{{{\text{1j}}}} = & { {\upgamma }}_{{10}} \\ { {\upbeta }}_{{{\text{2j}}}} = & { {\upgamma }}_{{20}} \\ \end{aligned}$$

$$\begin{gathered} {\text{with}} \hfill \\ { {\upvarepsilon }}_{{{\text{ij}}}} \sim {\text{ N}}(0,{ {\upsigma }}_{{{\text{ij}}}}^{2} ),{\text{residual variance}} \hfill \\ v_{{0{\text{j}}}} \sim {\text{ N}}(0,{ {\upsigma }}_{{{\text{0j}}}}^{2} ),{\text{random intercept for the participants}} \hfill \\ v_{{{\text{i}}0}} \sim {\text{ N}}(0,{ {\upsigma }}_{{{\text{i0}}}}^{2} ),{\text{random intercept for the stimuli}} \hfill \\ \end{gathered}$$

**Figure 5:**
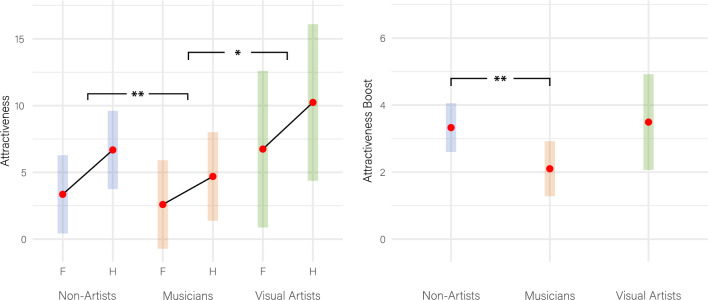
LMM results for Attractiveness ratings (cf. Supplementary Table S3). Left: EMMs including 95%-CI boundaries for facial portraits (F) and half-length torsos (H) across the three Status groups. The boosting effect is significantly steeper for both non-artists (**p < *0.01*) and visual artists (*p < *0.05*) as compared to musicians. Right: The boost in Attractiveness from torsos to facials is significantly lower (**p < *0.01*) in musicians when compared to non-artists. Due to the stricter testing approach in this analysis, the difference between visual artists and musicians is no longer significant (*p* = *0.067*), but it is still visible as a trend.

The results show two significant effects (Supplementary Table S3, Fig. 5 left): First, a main effect for the portrait’s Layout (*ß* = *3.327 [2.864, 3.789], t* = *14.110, p* < *0.00001, d* = *0.159*), which is the contextual enhancement effect we already know from the General Observations. Second, a significant interaction between Layout and Status, reflecting that musicians have a lower boost than both non-artists *(ß = −1.227 [−1.960,  − 0.492], t = −3.276, p = 0.00274, d = 0.059) and visual artists (ß = −1.392 [−2.706,  − 0.078], t = −2.077, p = 0.0378, d = 0.066)*. This interaction is of main interest for our hypothesis, as it indicates a moderating quality of the person’s artistic status on the strength of the contextual enhancement effect (even though, in the direction opposite as expected).

To directly compare the boosting effect among the three status groups (musicians, visual artists, non-artists), we conducted a second analysis in which the boost was predicted directly by the person’s status (Model-A-Boost). This model is identical to Model-A, with the exception that the factor Layout is dropped, as the difference between the layouts is expressed directly in the dependent variable Boost. This approach is stricter for testing the hypothesis due to the smaller variance in our difference scores when compared to the original values (*SD* = *16.056, SD* = *20.984*, respectively).

$${\text{Level}}\;1: \quad {\text{Boost}}_{{{\text{ij}}}} = {\upbeta }_{{0{\text{j}}}} + {\upbeta }_{{{\text{i}}0}} + {\upbeta }_{{2{\text{j}}}} {\text{Status}}_{{{\text{ij}}}} + {\upvarepsilon }_{{{\text{ij}}}}$$

$$\begin{aligned} {\text{Level}}\;{2}: \quad {\upbeta }_{{0{\text{j}}}} = & {\upgamma }_{00} + v_{{0{\text{j}}}} \\ {\upbeta }_{{{\text{i}}0}} = & {\upgamma }_{00} + v_{{{\text{i}}0}} \\ {\upbeta }_{{{\text{1j}}}} = & {\upgamma }_{{{1}0}} \\ {\upbeta }_{{{\text{2j}}}} = & {\upgamma }_{{{2}0}} \\ \end{aligned}$$

$$\begin{gathered} {\text{with}} \hfill \\ {\upvarepsilon }_{{{\text{ij}}}} \sim {\text{ N}}(0,{\upsigma }_{{{\text{ij}}}}^{2} ),{\text{residual variance}} \hfill \\ v_{{0{\text{j}}}} \sim {\text{ N}}(0,{\upsigma }_{{{\text{0j}}}}^{2} ),{\text{random intercept for the participants}} \hfill \\ v_{{{\text{i}}0}} \sim {\text{ N}}(0,{\upsigma }_{{{\text{i0}}}}^{2} ),{\text{random intercept for the stimuli}} \hfill \\ \end{gathered}$$

The results of Model-A-Boost (Supplementary Table S3, Fig. 5 right) show a main effect for Status (*F*_*2,187*_ = *4.646, p* = *0.011*), which is driven by the musicians’ lower boost when compared to non-artists (*ß* = *−1.226 [−2.052,  − 0.401], t* = *−2.907, p* = *0.004, d* = *0.076*).

**Figure 6:**
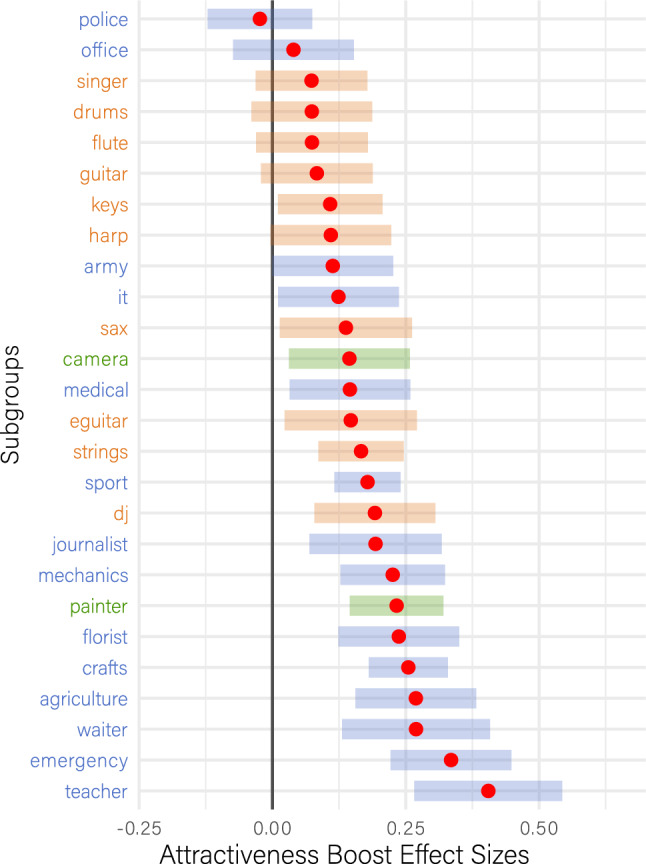
Effect sizes for attractiveness boosts per subgroup. The forest plot depicts EMMs for the attractiveness boosts, expressed as Cohen’s d for each stimulus and predicted in a linear model by subgroup membership. The error bars represent 95% confidence limits. For better readability, the superordinate status membership of the subgroups is color-coded: non-artists are shown in blue, musicians in orange and visual artists in green.

We further explored the boosting mechanism by examining the subgroups within each status group (cf. Table 1). To this end, we expressed the boost effect for each stimulus as Cohen’s d (by subtracting facial attractiveness ratings from torso ratings and dividing by the pooled standard deviation) and predicted these effect sizes in a linear regression through subgroup-membership. The results in Fig. 6 show that most subgroups within musicians are located at the top of the forest plot, indicating a small or nonexistent boosting effect. Conversely, most non-artistic subgroups tend to be located at the bottom of the forest plot (with the particular exception of police officers and office employees). For visual artists, painters had the greatest boost effect sizes.

### Gender effect

To test for a potential cross-gender effect, we extended Model-A-Boost by adding the interacting factors of the stimulus’ gender and the rater’s gender. In the outcomes of this model, the main effect of Status remained virtually identical compared to the original model (*F*_*2,187*_ = *4.671, p* = *0.011)*, whereas neither of the two additional gender factors nor their interaction term had any power to predict the boost effects (Supplementary Table S4a). Similarly, when testing the boost effect for a reduced data set, considering only ratings for opposite-sex faces, no effects compatible with our prediction were observed. Specifically, the additional models revealed no effects for females rating males (Supplementary Table S4b) and the previously observed disadvantage for both musicians and visual artists compared to non-artists when males were rating females (Supplementary Table S4c). The latter disadvantage is not specific to male raters, by the way; it is also observed for females rating females.

### Mean facial attractiveness

In this LMM, we predicted the attractiveness boost using the portrait’s mean facial attractiveness (computed from the entire sample) and the Status factor. The results show that neither mean facial attractiveness (*F*_*1,184*_ = 0.256*, p* = 0.614) nor its interaction with Status (*F*_*2,184*_ = *0.903, p* = 0.407) can predict the attractiveness boosts (Supplementary Table S5).

### Other variables

**Figure 7:**
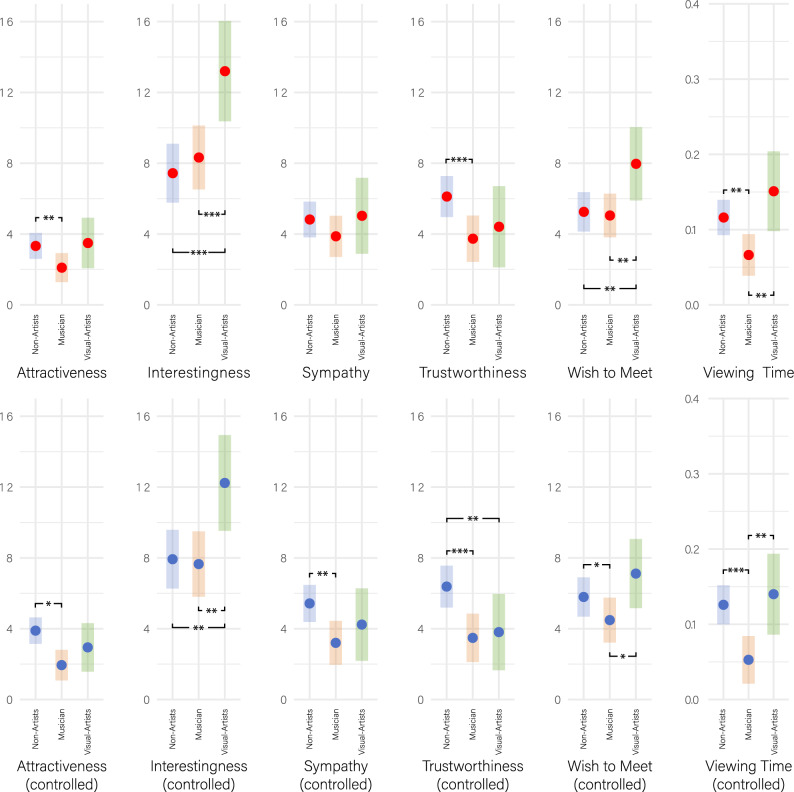
Status-specific boost effects for all scales and Viewing Time. Above: Boost effects across all scales for the three status groups (shown as EMMs including their 95%-CIs). Note that musicians have a lower boost than non-artists for Attractiveness, Trustworthiness and Viewing Time, with the latter also lower than visual artists. On the contrary, visual artists have a higher boost than both musicians and non-artists for Interestingness and Wish-to-Meet. Below: The same boost effects, but controlled for potential covariates arising from stimuli and raters. Note that the general result pattern prevails or becomes even more pronounced, making, for instance, the difference between musicians and non-artists on Sympathy now statistically significant. *p < 0.05, **p < 0.01, ***p < 0.001.

We fitted the same models as above for the ratings of the other four scales (Interestingness, Sympathy, Trustworthiness, and Wish-to-Meet) as well as the self-paced Viewing Time. Detailed results of these H2 analyses, along with the figures equivalent to Fig. 5 and 6 are given in the Supplementary Information (Supplementary Tables S6-S10 and Figs. S3-S8). Here, we present a summary of the modeling results for all variables (Fig. 7, above), for which we predicted the boost effects directly through the factor Status. In other words, similar to Model-A-Boost, we fitted a Model-I-Boost for Interestingness, a Model-S-Boost for Sympathy, etc. Accordingly, to test H3a (cross-gender effect) and H3b (mean facial value) for the other variables, we fitted equivalent models and present the results in the Supplementary Information (Supplementary Tables S11-S20). Collectively, we can summarize that H3a and H3b were also not supported for any of the other variables.

### Control for covariates

In the final step, we incorporated several covariates into the models to account for potential systematic differences that could arise from either the stimuli or the individual raters. Specifically, on the side of stimuli, we considered the following potential covariates:

1. Assumed age of the portrayed character. For this, ten independent raters (five females, five males) who were not part of the original sample estimated the person’s age on each portrait, and we averaged these ratings per stimulus.
2. Gender of the portrayed character.
3. Smile intensity of the portrayed character. We used intensity estimates of Action Unit 12 (AU12—lip corner puller, ranging between 0 and 4) from the Facial Action Coding System (FACS^40^).
4. Gaze direction of the portrayed character, either facing the viewer or looking aside. Both smile intensity and gaze direction were estimated by a trained FACS coder.
5. Socio-economic status of each profession appearing in the study. Occupations differ systematically in this regard, with specific professions, such as IT technicians or physicians, known to earn considerably more money than, for instance, artisans or musicians. For this covariate, we gathered estimated incomes for each professional subgroup from the German Employment Agency^41^. For the subgroups of musicians, we relied on data from the German Artists’ Social Security Fund^42^, the main insurance institution for artists in Germany.
6. Order in which each portrait appeared within the randomization for every participant.

On the side of the raters, we controlled for:

1. Their own gender.
2. Their own musical status: Information on how much they currently play or have ever played a musical instrument.
3. Their individual preference for, or liking of, the professions they have seen in the study, which was collected at the end of the second session (please see Procedure section).

The results of the models that included these covariates demonstrate predominantly their predictive power as main effects for Attractiveness (Supplementary Table S21). For instance, expectedly, estimated age of the depicted person has a negative impact on Attractiveness (*ß* = *−0.402 [−0.581,  − 0.222], t* = *−4.317, p* < *0.0001, d* = *0.02*) and Wish-to-Meet (*ß* = *−0.166 [−0.291,  − 0.042], t* = *−2.587, p* = *0.010467, d* = *0.008*). Conversely, personal preference for specific professions has a positive influence on all rating scales (although not on Viewing Time). Moreover, when comparing the models, it is evident that including the covariates significantly improves the statistical model fit, except for adding the gaze direction of the portrayed character, the rater’s own gender, and their own musical status (Supplementary Table S22). However, incorporating covariates does not alter the general result pattern (Fig. 7, below). On the contrary, controlling for the listed covariates results in an even more pronounced divergence of the boosts for musicians and non-artists, leading to significantly lower boosts for musicians on the Sympathy scale and Wish-to-Meet, which were previously only different by trend.

## Discussion

The quest for the historical origins of modern humans’ outer appearance and behavior has been central to the development of Darwin’s groundbreaking evolutionary theory. For human musical expression, Darwin proposed a mechanism that is both highly plausible and analogous to examples in the animal realm. If accurate, this mechanism should have left traces on a population-wide level in the form of an associative link between musicality and physical attractiveness. We investigated this link in our study by means of an implicit paradigm.

The results, however, point in the opposite direction of what we expected. Environmental cues that identified a person as a musician boosted their perceived attractiveness to a lesser degree than cues identifying a person as a teacher, waiter, farmer, artisan, or most other non-artistic control conditions in our study (Fig. 6). The same applies to almost all other measures of appeal: musicians had a lower boost in Sympathy, Trustworthiness, Wish-to-Meet and self-paced stimulus exploration time (Fig. 7), which we captured as an implicit measure of attentional binding and a character’s charming qualities. This result pattern was robust and, in fact, even more pronounced when accounting for potential confounding effects, whether arising from the stimuli or the individual raters, such as smile intensity of the portrayed character or the rater’s personal liking of the vocational specializations presented in this study.

The expected effects also did not emerge when testing in a cross-gender direction (e.g., male portraits assessed by female raters) or when considering the mean facial attractiveness of the portrayed character. Importantly, artists and non-artists did not differ on any of the scales used in our study, meaning that Status alone had no predictive power for any rating scale as a main effect (Supplementary Tables S3, S6-S9). The fact that no group was inherently more attractive, sympathetic, trustworthy, or interesting underscores our efforts to balance the stimuli across status groups prior to data collection.

Interestingly, when explicitly asked whether artists were more appealing (attractive, interesting, likeable, exciting, etc.) than non-artists, participants decided in favor of the artists on almost all scales (Fig. 3). Additionally, in absolute numbers, musicians and painters emerged as the most preferred professions, with average scores exceeding 70 on a 0 to 100 scale, while military personnel, police officers, and office employees ranked lowest in this regard, with scores falling below 30 points (thus fitting the outcomes of the implicit data in Fig. 6).

The discrepancy between the implicit outcomes and the conscious judgments is a puzzling inconsistency! It may reveal a general disparity in people’s explicitly stated attitudes towards artists compared to their unconscious dispositions. In other words, only the explicit (and publicly expressed) view of artists is highly appreciative. This aligns not only with Darwin’s reasoning but also with (Western) social conventions, which attribute high personal and societal value to the arts and those who practice them. Explicit reports can thus be subject to distortions by social desirability. On an implicit, unconscious level, however, our data suggest that musicians and visual artists are perceived as less attractive, less likeable, and also less reliable than non-artists.

The only exception is Interestingness for visual artists, which was the only outcome consistent with the sexual selection hypothesis (Fig. 7). While we are cautious not to over-interpret this result, it is noteworthy that Interestingness appears to behave fundamentally differently than all the other measures we applied in our study. Artists, particularly visual artists, seem to have an advantage on this scale, or at the very least, they exhibit no disadvantage as seen on all the other scales. Remarkably, this is also reflected in the explicit statement made by participants at the end of our study (Fig. 3). Here, artists were judged to be by far more interesting than non-artists. Interestingness could thus serve as a potential factor that prompts people to approach an artist in the first place. Subsequent (real-life) interactions could then lead to romantic attraction, provided that additional conditions are met.

We explored this idea by conducting a multilevel mediation analysis^43^ on our boost data, which we grand-mean-centered for this purpose (this analysis was exploratory and thus not pre-registered). First, we predicted Wish-to-Meet by Attractiveness (path c in mediation analysis terminology, with the former representing the dependent and the latter the independent variable). Unsurprisingly, this resulted in a positive association (*ß* = *0.521 [0.474, 0.565], t* = *22.52, p* < *0.001, d* = *0.03*). Next, we tested the link between Attractiveness and Interestingness (path a between the independent variable and the moderator). This also showed a significant effect: *ß* = *0.516 [0.467, 0.564], t* = *20.92, p* < *0.001, d* = *0.026*. In the last step, we predicted the effect of the mediator Interestingness on the dependent variable Wish-to-Meet (path b, *ß* = *0.486 [0.452, 0.519], t* = *28.75, p* < *0.001, d* = *0.027*), as well as the effect of Attractiveness on Wish-to-Meet when controlling for the mediator Interestingness (path c’, *ß* = *0.266 [0.233, 0.3], t* = *15.77, p* < *0.001, d* = *0.015*). The decrease in the effect of path c to path c’ (from 0.52 to 0.26, respectively) indicates that the link between Attractiveness and Wish-to-Meet is partially mediated by Interestingness. In other words, the desire to meet a depicted person is influenced to a substantial degree by attractiveness that is based on the interestingness this person radiates. Interestingness in turn is particularly pronounced for people who are artists, especially visual artists, as our data suggest (Fig. 7). Future studies could further explore this dual-step mechanism, in which perceived interestingness serves as the initiator to approach an artistic person.

The implicit design we employed to test Darwin’s hypothesis, providing the same face both as a facial portrait and a half-length portrait, revealed a general phenomenon that we term the ‘contextual enhancement effect.’ It suggests that individuals are generally viewed more favorably and capture longer spans of attention when portrayed in half-length portraits, which offer contextual information about their interests, rather than in just facial portraits. To our knowledge, this is a previously unreported phenomenon, although there is a substantial body of research on the influence of social contextualization. Specifically, the so-called ‘cheerleader effect’ refers to the tendency for people to rate individuals as more attractive when they are part of a group, compared to when they are viewed in isolation^44^. In our case, the context did not include other individuals but rather an everyday scenery that provided hints about the person’s vocational interests (cf. Figure 1).

How can the contextual enhancement effect be explained? While longer viewing times can be easily attributed to the fact that half-length portraits provide more visual information, it is more challenging to understand why individuals are also generally perceived as more attractive, likable, trustworthy, interesting, and more desirable to meet. In attempting to explain this effect, we would like to begin by noting that half-length portraits include the upper body of the depicted person, along with their posture. These are important cues for judging a person’s appearance. Furthermore, the everyday environment reveals not only the person’s interests, thus adding more individuality, but also conveys a primitive storyline, including the person’s role in this narrative. In our case, this narrative might show an individual appearing to be satisfied with, and confident in, their activities. All these elements are absent in facial portraits. Notably, in the case of police officers and office employees (professions that have been rated as the least liked), we observe that the contextual enhancement effect can be overridden by the raters’ general reluctance to a particular professional group. Here, half-length portraits received equal, and by tendency even lower, scores than facial portraits. The approach of cropping information on a person’s profession, interests, and surrounding environment could thus be useful in research contexts other than mate choice.

In conclusion, it is important to note that in this study, we tested Darwin’s hypothesis using a Western sample. It would be important to adapt and replicate our design with samples from other cultural backgrounds. Additionally, while we chose to use an implicit approach in this study, research designs that incorporate controlled use of confederates are particularly valuable. They would come closest to what Darwin had in mind when formulating his idea—an authentic multi-sensory real-life interaction between individuals. To our knowledge, studies of this nature have never been conducted. However, based on our current findings, we strongly encourage such future studies to include valid control conditions and not rely solely on explicit judgments.

Future research could also systematically manipulate the level of performative skill of an artist or musician, as the effect described by Darwin might be bound to outstanding display behavior (early results do suggest this direction^29^). Superior performance, in fact, is essential to the general mechanism of sexual selection. Only individuals with exceptional performative abilities (be it in music, ornamentation, courtship displays, aggression toward competitors, etc.) are likely to have the best chances of passing on their genes, thereby perpetuating the genetic basis of the very characteristics that enabled their exceptional performance in the first place. For musical displays, we would expect the effect to emerge more prominently in the case of highly skilled virtuosic musicians performing in front of an enthusiastic and admiring audience. Such scenarios are also likely to result in a perceivable boost in the person’s social standing. Conversely, poor performances that lead to reactions of embarrassment and disdain from the audience would likely have the opposite effect. In other words, we would not expect a positive effect from just any form of music making. The aspect of skill level was not the focus of our study. However, this applies to all specialization, including the non-artistic controls (crafts, mechanists, teachers, physicians, etc.), which can also systematically vary in terms of skill level. Since higher skill levels are likely to be preferred by participants in general, it will remain important for future studies to investigate the specificity of this variable’s impact on romantic attraction by separately examining artists and non-artists.

In addition to considering the skill level, future studies should also take into account the emotional impact on the perceiver. In his writings, Darwin emphasizes repeatedly the emotional power and seductiveness of music. The emotional impact might be another necessary component for the mechanism to be triggered.

### Supplementary Information

Supplementary Information.

## Data Availability

All preprocessed data and the scripts necessary to replicate the results and figures are available on the Open Science Framework (https://osf.io/9ypcq).
