# Supplementary material for: How being perceived to be an artist boosts feelings of attraction in others
Source: Sci Rep. 2023 Oct 31;13:18747. doi: 10.1038/s41598-023-45952-0 (PMC10618184; doi:10.1038/s41598-023-45952-0)
Supplement: Supplementary file 1 — Supplementary Information. [file 41598_2023_45952_MOESM1_ESM.pdf]

Supplementary Information

for the manuscript

“How being perceived to be an artist boosts feelings of attraction in others”

by

Eugen Wassiliwizky, Paul Wontorra, and Fredrik Ullén

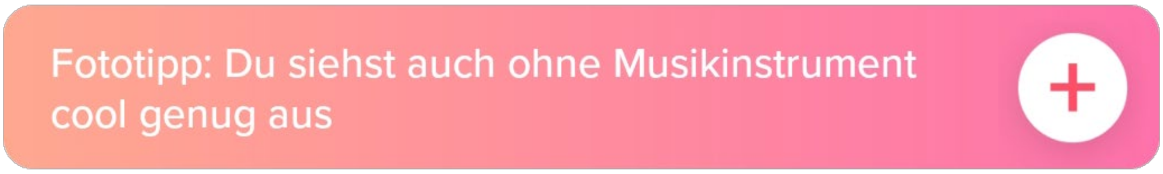

Figure S1. Screen shot from the dating platform *Tinder*. The German text reads: *Photo advice: You look cool enough without a musical instrument*.

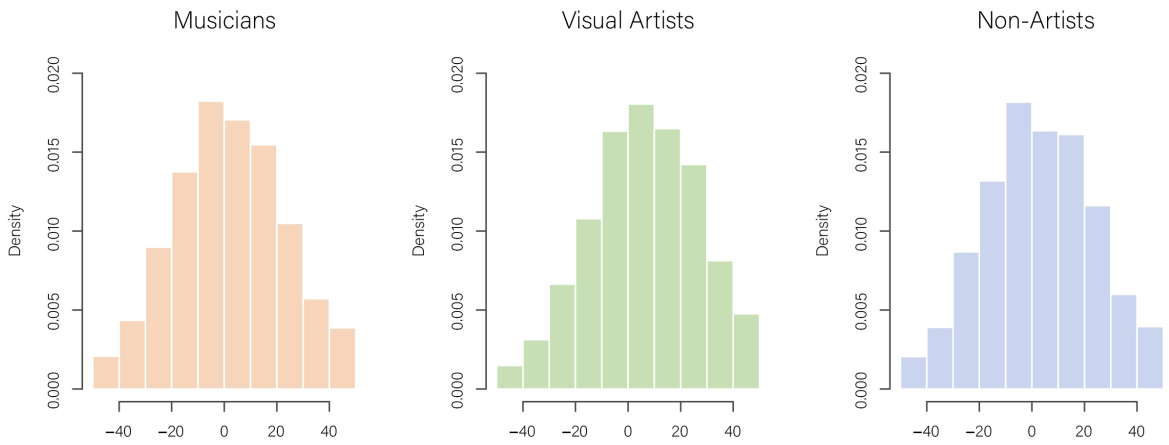

Figure S2. Density plots from the actual sample for facial attractiveness ratings of musicians, visual artists, and non-artists. Note that the collected data are (normally) distributed across the entire spectrum of the scale range, and that there is no systematic difference in attractiveness ratings between the three status groups. This is also reflected in statistical modeling (Model-A, Table S3), as Status per se does not predict Attractiveness ratings.

Table S1. General Observations I: Results for LMMs predicting ViewingTime (of faces and torsos) through mean facial attractiveness. The following model has been fitted:

Level 1: $\text{ViewingTime}_{ij} = \beta_{0j} + \beta_{i0} + \beta_{1j}\,\text{MeanFacialAttractiveness} + \varepsilon_{ij}$

	Faces			***Torsos***		
Predictors	*Estimates [95%-CI]*	*t*	*p*	*Estimates [95%-CI]*	*t*	*p*
**Fixed Effects**						
(Intercept)	9.865 [9.782, 9.949]	232.138	<0.001	9.967 [9.877, 10.056]	219.035	<0.001
MeanFA	**0.001 [0.001, 0.002]**	**2.657**	**0.0085**	**0.001 [0.000, 0.002]**	**2.164**	**0.0317**
**Random Part**						
σ^2	0.263			0.275		
$\tau_0{}^2$	0.002_{Stim}			0.006		
$\tau_0{}^2$	0.142_{Sub}			0.162		
ICC	0.353			0.379		
N	190_{Stim} / 80_{Sub}			190_{Stim} / 80_{Sub}		
Observations	15200			15200		
Conditional R^2 / Marginal R^2	0.354 / 0.0004			0.379 / 0.001		

Table S2. General Observations II: LMM results predicting Ratings of each scale through the portrait's Layout format. The following model has been fitted:

Level 1: $Rating_{ij} = \beta_{0j} + \beta_{i0} + \beta_{1j}\, Layout + \varepsilon_{ij}$

	Attractiveness			*Interestingness*			*Sympathy*		
Predictors	*Estimates [95%-CI]*	*t*	*p*	*Estimates [95%-CI]*	*t*	*p*	*Estimates [95%-CI]*	*t*	*p*
**Fixed Effects**									
(Intercept)	0.469 [-2.135, 3.073]	0.354	0.724	-7.648 [-10.271, -5.020]	-5.725	<0.001	2.245 [-0.270, 4.760]	1.754	0.081
Layout	**2.896 [2.552, 3.239]**	**16.516**	**<0.001**	**8.244 [7.836, 8.652]**	**39.581**	**<0.001**	**4.494 [4.142, 4.845]**	**25.056**	**<0.001**
**Random Part**									
σ^2	233.60			329.68			244.47		
τ_0^2	124.17_{Stim}			62.69_{Stim}			94.45_{Stim}		
τ_0^2	82.17_{Sub}			107.62_{Sub}			84.82_{Sub}		
ICC	0.469			0.341			0.423		
N	190_{Stim} / 80_{Sub}			190_{Stim} / 80_{Sub}			190_{Stim} / 80_{Sub}		
Observations	30400			30400			30400		
Conditional R^2 / Marginal R^2	0.472 / 0.005			0.362 / 0.033			0.430 / 0.012		

Table S2 [continued]. LMM results predicting Ratings through the portrait's Layout.

	Trustworthiness			***Wish-to-Meet***			***ViewingTime***		
Predictors	*Estimates [95%-CI]*	*t*	*p*	*Estimates [95%-CI]*	*t*	*p*	*Estimates [95%-CI]*	*t*	*p*
**Fixed Effects**									
(Intercept)	1.465 [-0.905, 3.835]	1.215	0.226	-7.009 [-9.659, -4.358]	-5.20	<0.001	9.764 [9.677, 9.853]	218.6 5	<0.001
Layout	**5.110 [4.756, 5.463]**	**28.323**	**<0.001**	**5.407 [5.025, 5.790]**	**27.71**	**<0.001**	**0.101 [0.089, 0.113]**	**16.89**	**<0.001**
**Random Part**									
σ^2	247.36			289.40			0.271		
$\tau_0{}^2$	56.66_{Stim}			65.71_{Stim}			0.151_{Stim}		
$\tau_0{}^2$	85.87_{Sub}			110.02_{Sub}			0.271_{Sub}		
ICC	0.366			0.378			0.362		
N	190_{Stim} / 80_{Sub}			190_{Stim} / 80_{Sub}			190_{Stim} / 80_{Sub}		
Observations	30400			30400			30400		
Conditional R^2 / Marginal R^2	0.376 / 0.016			0.387 / 0.015			0.366 / 0.006		

Table S3. LMM results predicting Attractiveness through the portrait's Layout (face, torso) and Status of the depicted person (musician, visual artist, non-artist), as well as the Attractiveness-Boost just through Status. To test H2, the following models have been fitted:

Model-A: Level 1: $\text{Attractiveness}_{ij} = \beta_{0j} + \beta_{i0} + \beta_{1j}\ \text{Layout}_{ij} * \beta_{2j}\ \text{Status}_{ij} + \varepsilon_{ij}$

Model-A-Boost: Level 1: $\text{Attractiveness-Boost}_{ij} = \beta_{0j} + \beta_{i0} + \beta_{1j}\ \text{Status}_{ij} + \varepsilon_{ij}$

	Attractiveness (Model-A)			***Attractiveness-Boost (Model-A-Boost)***		
Predictors	*Estimates [95%-CI]*	*t*	*p*	*Estimates [95%-CI]*	*t*	*p*
**Fixed Effects**						
(Intercept)	0.0285 [-2.969, 3.026]	0.019	0.985	3.327 [2.6, 4.05]	8.968	<0.001
Layout	**3.327 [2.864, 3.789]**	**14.11**	**<0.001**			
Status: M<Non	0.465 [-3.102, 4.033]	0.255	0.798	**-1.226 [-2.053, -0.401]**	**2.907**	**0.004**
Status: V<Non	3.222 [-2.956, 9.402]	1.021	0.308	0.165 [-1.264, 1.596]	0.227	0.821
Status: M<V	-2.757 [-9.146, 3.631]	-0.845	0.399	-1.392 [-2.871, 0.0865]	-1.843	0.067
L*S: M<Non	**-1.227 [-1.961, -0.493]**	**-3.276**	**0.001**			
L*S: V<Non	0.166 [-1.105, 1.437]	0.256	0.798			
L*S: M<V	**-1.392 [-2.707, -0.079]**	**-2.077**	**0.038**			
**Random Part**						
σ^2	233.52			247.942		
τ_0^2	123.83_{Stim}			4.316_{Stim}		
τ_0^2	82.17_{Sub}			5.362_{Sub}		
ICC	0.469			0.038		
N	190_{Stim} / 80 $_{Sub}$			190_{Stim} / 80 $_{Sub}$		
Observations	30400			15200		
Conditional R^2 / Marginal R^2	0.473 / 0.009			0.039 / 0.001		

Table S4a. LMM results predicting Attractiveness-Boost through Status of the depicted person (musician, visual artist, non-artist) and the stimulus' gender (male, female) interacting with the rater's gender (male, female). To test H3a, the following model has been fitted:

Level 1: $AttractivenessBoost_{ij} = \beta_{0j} + \beta_{i0} + \beta_{1j}\ Status_{ij} + \beta_{2j}\ Stim\_Gender_{ij} * \beta_{3j}\ Rater\_Gender_{ij} + \varepsilon_{ij}$

	AttractivenessBoost		
Predictors	*Estimates [95%-CI]*	*t*	*p*
**Fixed Effects**			
(Intercept)	3.232 [2.181, 4.284]	5.997	<0.001
Status: M<Non	**-1.222 [-2.042, -0.402]**	**-2.911**	**0.004**
Status: V<Non	0.176 [-1.244, 1.595]	0.241	0.809
Status: M<V	-1.398 [-2.865, 0.07]	-1.859	0.065
Stim_Gen: m<f	0.441 [-0.489, 1.371]	0.927	0.355
Rater_Gen: m<f	-0.482 [-1.729, 0.765]	-0.756	0.451
S_Gen*R_Gen: m<f	0.447 [-0.556, 1.45]	0.874	0.382
**Random Part**			
σ^2	247.946		
τ_0^2	4.239_{Stim}		
τ_0^2	5.431_{Sub}		
ICC	0.038		
N	190_{Stim} / 80_{Sub}		
Observations	15200		
Conditional R^2 / Marginal R^2	0.039 / 0.002		

Table S4b. LMM results for a reduced data set of female raters and male portraits, predicting Attractiveness-Boost through Status of the depicted person (musician, visual artist, non-artist). This is thus a modified version of Model-A-Boost for a reduced data set:

Level 1: $\text{Attractiveness-Boost}_{ij} = \beta_{0j} + \beta_{i0} + \beta_{1j}\ \text{Status}_{ij} + \varepsilon_{ij}$

	AttractivenessBoost		
Predictors	*Estimates [95%-CI]*	*t*	*p*
**Fixed Effects**			
(Intercept)	3.668 [2.490, 4.884]	6.092	<0.001
Status: M<Non	-1.153 [-2.631, 0.324]	-1.525	0.131
Status: V<Non	-0.471 [-3.05, 2.108]	-0.357	0.722
Status: M<V	-0.682 [-3.35, 1.986]	-0.5	0.618
**Random Part**			
σ^2	287.413		
τ_0^2	5.296_{Stim}		
τ_0^2	5.779_{Sub}		
ICC	0.037		
N	97_{Stim} / 42_{Sub}		
Observations	4074		
Conditional R^2 / Marginal R^2	0.038 / 0.001		

Table S4c. LMM results for a reduced data set of male raters and female portraits, predicting Attractiveness-Boost through Status of the depicted person (musician, visual artist, non-artist). This is thus a modified version of Model-A-Boost for a reduced data set:

Level 1: $\text{Attractiveness-Boost}_{ij} = \beta_{0j} + \beta_{i0} + \beta_{1j}\ \text{Status}_{ij} + \varepsilon_{ij}$

	AttractivenessBoost		
Predictors	*Estimates [95%-CI]*	*t*	*p*
**Fixed Effects**			
(Intercept)	3.424 [2.205, 4.642]	5.504	<0.001
Status: M<Non	**-1.96 [-3.348, -0.573]**	**-1.525**	**0.007**
Status: V<Non	1.086 [-1.297, 3.469]	0.891	0.375
Status: M<V	**-3.046 [-5.509, -0.584]**	**-2.418**	**0.018**
**Random Part**			
σ^2	217.494		
τ_0^2	4.552_{Stim}		
τ_0^2	7.052_{Sub}		
ICC	0.051		
N	93_{Stim} / 38_{Sub}		
Observations	3534		
Conditional R^2 / Marginal R^2	0.055 / 0.005		

Table S5. LMM results predicting Attractiveness-Boost through mean facial attractiveness interacting with the Status of the depicted person (musician, visual artist, non-artist). To test H3b, the following model has been fitted:

Level 1: $\text{Attractiveness-Boost}_{ij} = \beta_{0j} + \beta_{i0} + \beta_{1j}\,\text{MeanFacialAttractiveness}_{ij} * \beta_{2j}\,\text{Status}_{ij} + \varepsilon_{ij}$

	Attractiveness-Boost		
Predictors	*Estimates [95%-CI]*	*t*	*p*
**Fixed Effects**			
(Intercept)	3.386 [2.647, 4.125]	8.941	<0.001
MeanFacialAttract	-0.017 [-0.063, 0.028]	-0.745	0.457
**Status: M<Non**	**-1.146 [-1.989, -0.302]**	**-2.637**	**0.009**
Status: V<Non	-0.112 [-1.787, 1.562]	-0.13	0.897
Status: M<V	-1.034 [-2.748, 0.681]	-1.171	0.243
MFA*S: M<Non	-0.036 [-0.109, 0.036]	-0.976	0.33
MFA*S: V<Non	0.05 [-0.089, 0.189]	0.7	0.485
MFA*S: M<V	-0.086 [-0.229, 0.056]	-1.177	0.241
**Random Part**			
σ^2	247.942		
$\tau_0{}^2$	4.266_{Stim}		
$\tau_0{}^2$	5.362_{Sub}		
ICC	0.037		
N	190_{Stim} / 80_{Sub}		
Observations	15200		
Conditional R^2 / Marginal R^2	0.039 / 0.002		

Table S6. LMM results predicting Interestingness through the portrait's Layout (face, torso) and Status of the depicted person (musician, visual artist, non-artist) as well as the Interestingness-Boost just through Status. To test H2, the following models have been fitted:

Model-I: Level 1: $\text{Interestingness}_{ij} = \beta_{0j} + \beta_{i0} + \beta_{1j}\ \text{Layout}_{ij} * \beta_{2j}\ \text{Status}_{ij} + \varepsilon_{ij}$

Model-I-Boost: Level 1: $\text{Interestingness-Boost}_{ij} = \beta_{0j} + \beta_{i0} + \beta_{1j}\ \text{Status}_{ij} + \varepsilon_{ij}$

	Interestingness (Model-I)			***Interestingness-Boost (Model-I-Boost)***		
Predictors	*Estimates [95%-CI]*	*t*	*p*	*Estimates [95%-CI]*	*t*	*p*
**Fixed Effects**						
(Intercept)	-8.123 [-10.962, -5.284]	-5.611	<0.001	7.437 [5.772, 9.102]	8.763	<0.001
**Layout**	**7.437 [6.889, 7.986]**	**26.569**	**<0.001**			
Status: M<Non	1.558 [-1.134, 4.251]	1.133	0.258	0.885 [-0.657, 2.428]	1.124	0.263
Status: V>Non	-1.052 [-5.715, 4.251]	-0.441	0.659	**5.760 [3.089, 8.432]**	**4.222**	**<0.001**
Status: M<V	2.610 [-2.211, 7.432]	1.059	0.290	**-4.875 [-7.637, -2.113]**	**-3.456**	**<0.001**
**L*S: M>Non**	**0.885 [0.014, 1.756]**	**1.992**	**0.046**			
**L*S: V>Non**	**5.760 [4.252, 7.269]**	**7.483**	**<0.001**			
**L*S: M<V**	**-4.875 [-6.435, -3.315]**	**-6.125**	**<0.001**			
**Random Part**						
σ^2	329.09			436.76		
τ_0^2	58.27_{Stim}			20.38_{Stim}		
τ_0^2	107.62_{Sub}			37.93_{Sub}		
ICC	0.335			0.118		
N	190_{Stim} / 80_{Sub}			190_{Stim} / 80_{Sub}		
Observations	30400			15200		
Conditional R^2 / Marginal R^2	0.364 / 0.044			0.122 / 0.005		

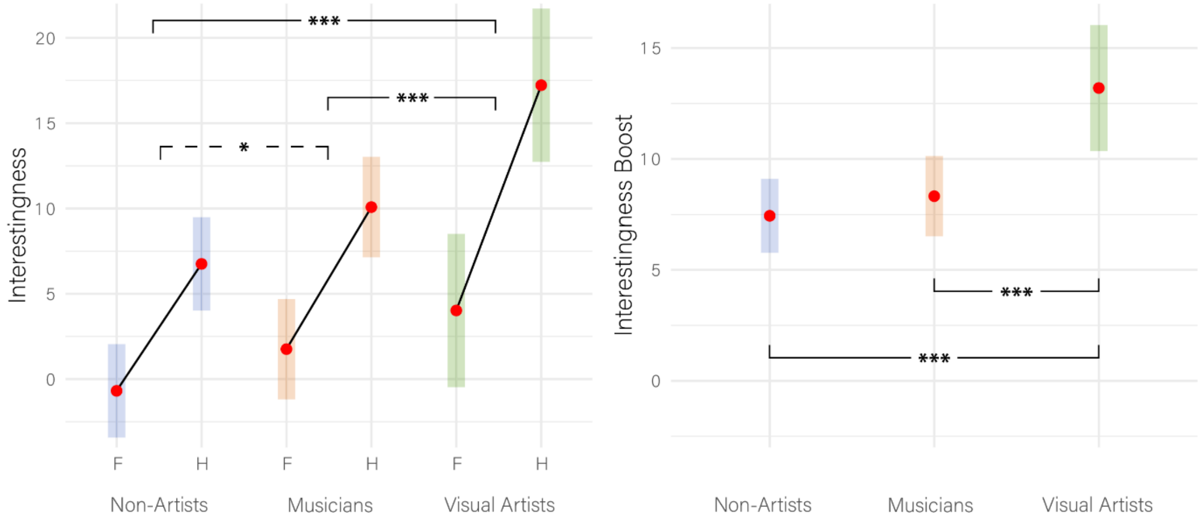

Figure S3. LMM results for Interestingness ratings (cf. Table S6). Left: EMMs including 95 % CI boundaries for facial portraits (F) and half-length torsos (H) across the three Status groups. The boosting effect is significantly steeper for both musicians (**p* < *0.05*) and visual artists (*****p* < *0.001*) as compared to non-artists. Among each other, the visual artists' boost is steeper than the boost of musicians (*****p* < *0.001*). Right: The boost in Interestingness from torsos to facials is significantly higher for visuals artists as compared to both musicians and non-artists (*****p* < *0.001*). Due to the stricter way of testing in this approach, the difference between musicians and non-artists is no longer significant (*p* = *0.263*), but only visible by trend.

Table S7. LMM results predicting Sympathy through the portrait's Layout (face, torso) and Status of the depicted person (musician, visual artist, non-artist) as well as the Sympathy-Boost just through Status. To test H2, the following models have been fitted:

Model-S: Level 1: $Sympathy_{ij} = \beta_{0j} + \beta_{i0} + \beta_{1j}\ Layout_{ij} * \beta_{2j}\ Status_{ij} + \varepsilon_{ij}$

Model-S-Boost: Level 1: $Sympathy\text{-}Boost_{ij} = \beta_{0j} + \beta_{i0} + \beta_{1j}\ Status_{ij} + \varepsilon_{ij}$

	Sympathy (Model-S)			*Sympathy-Boost (Model-S-Boost)*		
Predictors	*Estimates [95%-CI]*	*t*	*p*	*Estimates [95%-CI]*	*t*	*p*
**Fixed Effects**						
(Intercept)	1.893 [-0.951, 4.737]	1.303	0.194	4.821 [3.813, 5.828]	9.361	<0.001
**Layout**	**4.820 [4.348, 5.293]**	**19.982**	**<0.001**			
Status: M<Non	0.580 [-2.603, 3.763]	0.357	0.722	-0.948 [-2.220, 0.324]	-1.459	0.146
Status: V<Non	1.676 [-3.836, 7.189]	0.595	0.552	0.209 [-1.993, 2.411]	0.186	0.853
Status: M<V	-1.096 [-6.796, 4.603]	-0.376	0.707	-1.157 [-3.434, 1.120]	-0.994	0.321
**L*S: M<Non**	**-0.948 [-1.699, -0.197]**	**-2.474**	**0.013**			
L*S: V>Non	0.209 [-1.091, 1.509]	0.315	0.752			
L*S: M<V	-1.157 [-2.501, 0.187]	-1.687	0.092			
**Random Part**						
σ^2	244.43			301.236		
τ_0^2	94.90_{Stim}			13.818_{Stim}		
τ_0^2	84.82_{Sub}			7.817_{Sub}		
ICC	0.424			0.067		
N	190_{Stim} / 80_{Sub}			190_{Stim} / 80_{Sub}		
Observations	30400			15200		
Conditional R^2 / Marginal R^2	0.431 / 0.013			0.068 / 0.001		

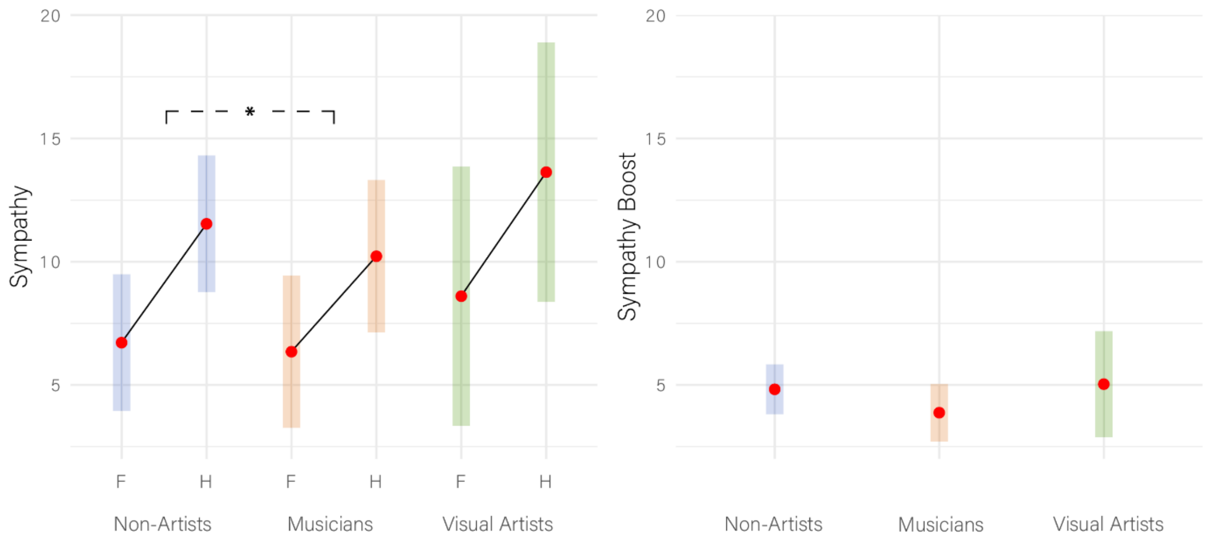

Figure S4. LMM results for Sympathy ratings (cf. Table S7). Left: EMMs including 95 % CI boundaries for facial portraits (F) and half-length torsos (H) across the three Status groups. The boosting effect is significantly steeper for non-artists as compared to musicians (**p* < *0.05*). Right: The boost in Sympathy from torsos to facials is not significantly different between the three status groups. Due to the stricter way of testing in this approach, the difference between musicians and non-artists is no longer significant (*p = 0.146*), but only visible by trend.

Table S8. LMM results predicting Trustworthiness through the portrait's Layout (face, torso) and Status of the depicted person (musician, visual artist, non-artist) as well as the Trustworthiness-Boost just through Status. To test H2, the following models have been fitted:

Model-T: Level 1: $\text{Trustworthiness}_{ij} = \beta_{0j} + \beta_{i0} + \beta_{1j}\ \text{Layout}_{ij} * \beta_{2j}\ \text{Status}_{ij} + \varepsilon_{ij}$

Model-T-Boost: Level 1: $\text{Trustworthiness-Boost}_{ij} = \beta_{0j} + \beta_{i0} + \beta_{1j}\ \text{Status}_{ij} + \varepsilon_{ij}$

	Trustworthiness (Model-T)			***Trustworthiness-Boost (Model-T-Boost)***		
Predictors	*Estimates [95%-CI]*	*t*	*p*	*Estimates [95%-CI]*	*t*	*p*
**Fixed Effects**						
(Intercept)	0.513 [-2.084, 3.110]	0.388	0.699	6.118 [4.963, 7.274]	10.367	<0.001
**Layout**	**6.118 [5.643, 6.594]**	**25.226**	**<0.001**			
Status: M<Non	1.682 [-0.884, 4.247]	1.283	0.201	**-2.381 [-3.714, -1.049]**	**-3.499**	**<0.001**
Status: V<Non	4.048 [-0.394, 8.491]	1.783	0.076	-1.706 [-4.013, 0.602]	-1.447	0.150
Status: M<V	-2.367 [-6.960, 2.227]	-1.008	0.314	-0.676 [-3.061, 1.710]	-0.554	0.580
**L*S: M<Non**	**-2.381 [-3.136, -1.627]**	**-6.183**	**<0.001**			
**L*S: V<Non**	**-1.706 [-3.013, -0.399]**	**-2.557**	**0.011**			
L*S: M<V	-0.676 [-2.027, 0.676]	-0.980	0.327			
**Random Part**						
σ^2	247.05			17.983		
τ_0^2	56.10_{Stim}			15.25_{Stim}		
τ_0^2	85.87_{Sub}			13.17_{Sub}		
ICC	0.365			0.081		
N	190_{Stim} / 80_{Sub}			190_{Stim} / 80_{Sub}		
Observations	30400			15200		
Conditional R^2 / Marginal R^2	0.378 / 0.020			0.084 / 0.004		

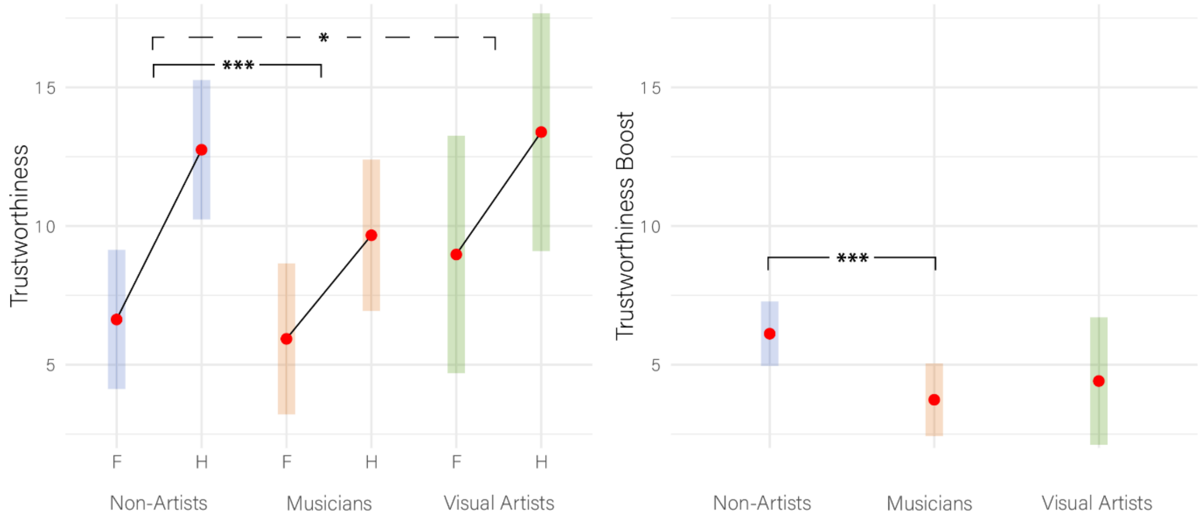

Figure S5. LMM results for Trustworthiness ratings (cf. Table S8). Left: EMMs including 95 % CI boundaries for facial portraits (F) and half-length torsos (H) across the three Status groups. The boosting effect of non-artists is significantly steeper than that of musicians (***$p < 0.001$) and visual artists (*$p < 0.05$). Right: The boost in Trustworthiness from torsos to facials is significantly higher for non-artists as compared to musicians (***$p < 0.001$). Due to the stricter way of testing in this approach, the difference between non-artists and visuals artists is no longer significant ($p = 0.15$), but only visible by trend.

Table S9. LMM results predicting Wish-to-Meet through the portrait's Layout (face, torso) and Status of the depicted person (musician, visual artist, non-artist) as well as the Wish-to-Meet-Boost just through Status. To test H2, the following models have been fitted:

Model-M: Level 1: $\text{Wish-to-Meet}_{ij} = \beta_{0j} + \beta_{i0} + \beta_{1j}\ \text{Layout}_{ij} * \beta_{2j}\ \text{Status}_{ij} + \varepsilon_{ij}$

Model-M-Boost: Level 1: $\text{Wish-to-Meet-Boost}_{ij} = \beta_{0j} + \beta_{i0} + \beta_{1j}\ \text{Status}_{ij} + \varepsilon_{ij}$

	Wish-to-Meet (Model-M)			***Wish-to-Meet-Boost (Model-M-Boost)***		
Predictors	*Estimates [95%-CI]*	*t*	*p*	*Estimates [95%-CI]*	*t*	*p*
**Fixed Effects**						
(Intercept)	-7.435 [-10.315, -4.556]	-5.064	<0.001	5.254 [4.142, 6.365]	9.264	<0.001
**Layout**	**5.254 [4.739, 5.768]**	**20.019**	**<0.001**			
Status: M<Non	0.775 [-1.972, 3.521]	0.552	0.581	-0.206 [-1.381, 0.968]	-0.344	0.731
Status: V>Non	1.727 [-3.030, 6.483]	0.711	0.478	**2.712 [0.678, 4.746]**	**2.610**	**0.010**
Status: M<V	-0.952 [-5.870, 3.965]	-0.379	0.705	**-2.918 [-5.021, -0.815]**	**-2.717**	**0.007**
L*S: M<Non	-0.206 [-1.023, 0.610]	-0.495	0.620			
**L*S: V>Non**	**2.720 [1.297, 4.126]**	**3.758**	**<0.001**			
**L*S: M<V**	**-2.918 [-4.381, -1.456]**	**-3.911**	**<0.001**			
**Random Part**						
σ^2	289.27			354.29		
τ_0^2	63.91_{Stim}			10.56_{Stim}		
τ_0^2	110.02_{Sub}			14.31_{Sub}		
ICC	0.376			0.081		
N	190_{Stim} / 80_{Sub}			190_{Stim} / 80_{Sub}		
Observations	30400			15200		
Conditional R^2 / Marginal R^2	0.389 / 0.021			0.084 / 0.004		

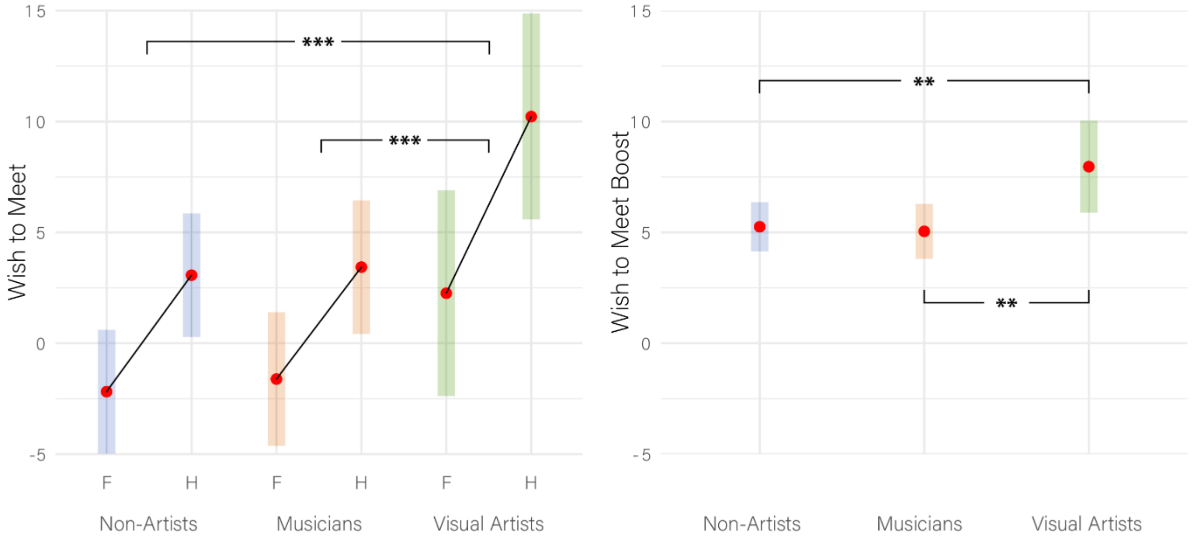

Figure S6. LMM results for Wish-to-Meet ratings (cf. Table S9). Left: EMMs including 95 % CI boundaries for facial portraits (F) and half-length torsos (H) across the three Status groups. The boosting effect of visual artists is significantly steeper than that for musicians and non-artists (***$p < 0.001$). Right: The same result pattern is reflected in the Model-M-Boost: The boost in Wish-to-Meet from torsos to facials is significantly higher for visual artists than for both non-artists and musicians (**$p < 0.01$).

Table S10. LMM results predicting ViewingTime through the portrait's Layout (face, torso) and Status of the depicted person (musician, visual artist, non-artist) as well as the ViewingTime-Boost just through Status. To test H2, the following models have been fitted:

Model-V: Level 1: $ViewingTime_{ij} = \beta_{0j} + \beta_{i0} + \beta_{1j}\ Layout_{ij} * \beta_{2j}\ Status_{ij} + \varepsilon_{ij}$

Model-V-Boost: Level 1: $ViewingTime\text{-}Boost_{ij} = \beta_{0j} + \beta_{i0} + \beta_{1j}\ Status_{ij} + \varepsilon_{ij}$

	ViewingTime (Model-V)			*ViewingTime-Boost (Model-V-Boost)*		
Predictors	*Estimates [95%-CI]*	*t*	*p*	*Estimates [95%-CI]*	*t*	*p*
**Fixed Effects**						
(Intercept)	9.745 [9.655, 9.835]	213.768	<0.001	0.116 [0.093, 0.140]	9.687	<0.001
**Layout**	**0.116 [0.100, 0.132]**	**14.442**	**<0.001**			
**Status: M<Non**	**0.062 [0.019, 0.104]**	**2.827**	**0.005**	**-0.050 [-0.082, -0.018]**	**-3.047**	**0.003**
Status: V<Non	-0.032 [-0.106, 0.042]	-0.856	0.392	0.035 [-0.020, 0.090]	1.234	0.219
**Status: M<V**	**0.094 [0.017, 0.170]**	**2.406**	**0.016**	**-0.085 [-0.142, -0.027]**	**-2.896**	**0.004**
**L*S: M<Non**	**-0.049 [-0.075, -0.025]**	**-3.901**	**<0.001**			
L*S: V<Non	0.035 [-0.008, 0.078]	1.580	0.114			
**L*S: M<V**	**-0.085 [-0.129, -0.039]**	**-3.707**	**<0.001**			
**Random Part**						
σ^2	0.271			0.554		
τ_0^2	0.003_{Stim}			0.004_{Stim}		
τ_0^2	0.151_{Sub}			0.003_{Sub}		
ICC	0.362			0.013		
N	190_{Stim} / 80_{Sub}			190_{Stim} / 80_{Sub}		
Observations	30400			15200		
Conditional R^2 / Marginal R^2	0.366 / 0.007			0.014 / 0.001		

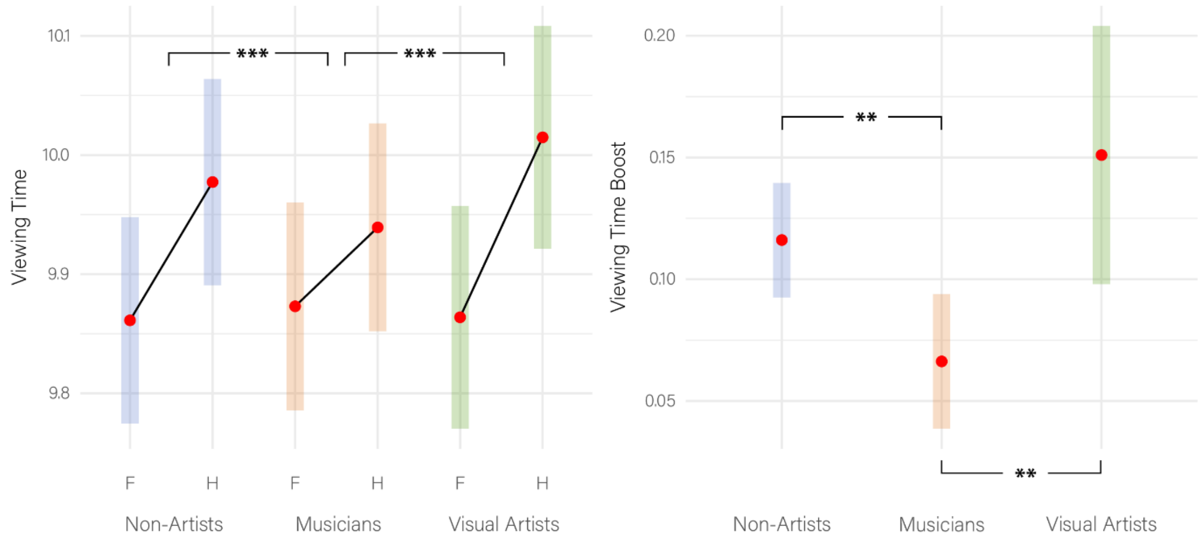

Figure S7. LMM results for ViewingTime (cf. Table S10). Left: EMMs including 95 % CI boundaries for facial portraits (F) and half-length torsos (H) across the three Status groups. The boosting effect is significantly flatter for musicians as compared to both non-artists and visual artists (***p < *0.001*). Right: The same result pattern is reflected in the Model-V-Boost: The boost in ViewingTime from torsos to facials is significantly lower for musicians as compared to the other two Status groups (**p < *0.01*).

Table S11. LMM results predicting Interestingness-Boost through Status of the depicted person (musician, visual artist, non-artist) and the stimulus' gender (male, female) interacting with the rater's gender (male, female). To test H3a, the following model has been fitted:

Level 1: $\text{Interestingness-Boost}_{ij} = \beta_{0j} + \beta_{i0} + \beta_{1j}\ \text{Status}_{ij} + \beta_{2j}\ \text{Stim\_Gender}_{ij} * \beta_{3j}\ \text{Rater\_Gender}_{ij} + \varepsilon_{ij}$

	Interestingness-Boost		
Predictors	*Estimates [95%-CI]*	*t*	*p*
**Fixed Effects**			
(Intercept)	6.751 [4.388, 9.113]	5.578	<0.001
Status: M<Non	0.891 [-0.646, 2.429]	1.133	0.259
**Status: V>Non**	**5.773 [3.110, 8.435]**	**4.235**	**<0.001**
**Status: M<V**	**-4.881 [-7.634, -2.129]**	**-3.464**	**<0.001**
Stim_Gen: m<f	0.833 [-0.766, 2.432]	1.018	0.310
Rater_Gen: m<f	0.471 [-2.409, 3.351]	0.320	0.749
S_Gen*R_Gen: m<f	0.040 [-1.291, 1.371]	0.060	0.952
**Random Part**			
σ^2	436.79		
τ_0^2	20.33_{Stim}		
τ_0^2	38.39_{Sub}		
ICC	0.119		
N	190_{Stim} / 80_{Sub}		
Observations	15200		
Conditional R^2 / Marginal R^2	0.123 / 0.005		

Table S12. LMM results predicting Sympathy-Boost through Status of the depicted person (musician, visual artist, non-artist) and the stimulus' gender (male, female) interacting with the rater's gender (male, female). To test H3a, the following model has been fitted:

Level 1: $\text{Sympathy-Boost}_{ij} = \beta_{0j} + \beta_{i0} + \beta_{1j}\ \text{Status}_{ij} + \beta_{2j}\ \text{Stim\_Gender}_{ij} * \beta_{3j}\ \text{Rater\_Gender}_{ij} + \varepsilon_{ij}$

	Sympathy-Boost		
Predictors	*Estimates [95%-CI]*	*t*	*p*
**Fixed Effects**			
(Intercept)	3.981 [2.583, 5.379]	5.550	<0.001
Status: M<Non	-0.940 [-2.199, 0.320]	-1.456	0.147
Status: V<Non	0.226 [-1.956, 2.407]	0.202	0.840
Status: M<V	-1.165 [-3.421, 1.090]	-1.008	0.315
Stim_Gen: m<f	1.243 [-0.071, 2.556]	1.846	0.066
Rater_Gen: m<f	0.480 [-0.985, 1.944]	0.642	0.522
S_Gen*R_Gen: m<f	-0.190 [-1.296, 0.915]	-0.338	0.736
**Random Part**			
σ^2	301.25		
$\tau_0{}^2$	13.58_{Stim}		
$\tau_0{}^2$	7.90_{Sub}		
ICC	0.067		
N	190_{Stim} / 80_{Sub}		
Observations	15200		
Conditional R^2 / Marginal R^2	0.068 / 0.002		

Table S13. LMM results predicting Trustworthiness-Boost through Status of the depicted person (musician, visual artist, non-artist) and the stimulus' gender (male, female) interacting with the rater's gender (male, female). To test H3a, the following model has been fitted:

Level 1: $\text{Trustworthiness-Boost}_{ij} = \beta_{0j} + \beta_{i0} + \beta_{1j}\ \text{Status}_{ij} + \beta_{2j}\ \text{Stim\_Gender}_{ij} * \beta_{3j}\ \text{Rater\_Gender}_{ij} + \varepsilon_{ij}$

	Trustworthiness-Boost		
Predictors	*Estimates [95%-CI]*	*t*	*p*
**Fixed Effects**			
(Intercept)	5.234 [3.630, 6.838]	6.364	<0.001
**Status: M<Non**	**-2.368 [-3.666, -1.069]**	**-3.561**	**<0.001**
Status: V<Non	-1.678 [-3.926, 0.570]	-1.457	0.147
Status: M<V	-0.690 [-3.014, 1.634]	-0.580	0.563
**Stim_Gen: m<f**	**-2.112 [-0.756, -3.467]**	**-3.042**	**0.003**
Rater_Gen: m<f	-0.240 [-2.039, 1.558]	-0.262	0.794
S_Gen*R_Gen: m<f	-0.281 [-1.426, 0.864]	-0.481	0.630
**Random Part**			
σ^2	323.39		
τ_0^2	14.37_{Stim}		
τ_0^2	13.32_{Sub}		
ICC	0.079		
N	190_{Stim} / 80_{Sub}		
Observations	15200		
Conditional R^2 / Marginal R^2	0.085 / 0.006		

Table S14. LMM results predicting Wish-to-Meet-Boost through Status of the depicted person (musician, visual artist, non-artist) and the stimulus' gender (male, female) interacting with the rater's gender (male, female). To test H3a, the following model has been fitted:

Level 1: $\text{Wish-to-Meet-Boost}_{ij} = \beta_{0j} + \beta_{i0} + \beta_{1j}\ \text{Status}_{ij} + \beta_{2j}\ \text{Stim\_Gender}_{ij} * \beta_{3j}\ \text{Rater\_Gender}_{ij} + \varepsilon_{ij}$

	Wish-to-Meet-Boost		
Predictors	*Estimates [95%-CI]*	*t*	*p*
**Fixed Effects**			
(Intercept)	4.755 [3.173, 6.338]	5.862	<0.001
Status: M<Non	-0.199 [-1.364, 0.965]	-0.334	0.739
**Status: V>Non**	**2.726 [0.709, 4.743]**	**2.639**	**0.009**
**Status: M<V**	**-2.926 [-5.011, -0.840]**	**-2.739**	**0.007**
Stim_Gen: m<f	1.166 [-0.092, 2.424]	1.810	0.071
Rater_Gen: m<f	-0.027 [-1.904, 1.850]	-0.028	0.977
S_Gen*R_Gen: m<f	-0.322 [-1.521, 0.876]	-0.527	0.598
**Random Part**			
σ^2	354.31		
τ_0^2	10.38_{Stim}		
τ_0^2	14.51_{Sub}		
ICC	0.066		
N	190_{Stim} / 80_{Sub}		
Observations	15200		
Conditional R^2 / Marginal R^2	0.068 / 0.002		

Table S15. LMM results predicting ViewingTime-Boost through Status of the depicted person (musician, visual artist, non-artist) and the stimulus' gender (male, female) interacting with the rater's gender (male, female). To test H3a, the following model has been fitted:

Level 1: $\text{ViewingTime-Boost}_{ij} = \beta_{0j} + \beta_{i0} + \beta_{1j}\ \text{Status}_{ij} + \beta_{2j}\ \text{Stim\_Gender}_{ij} * \beta_{3j}\ \text{Rater\_Gender}_{ij} + \varepsilon_{ij}$

	ViewingTime-Boost		
Predictors	*Estimates [95%-CI]*	*t*	*p*
**Fixed Effects**			
(Intercept)	0.118 [0.082, 0.153]	6.468	<0.001
**Status: M<Non**	**-0.050 [-0.082, -0.018]**	**-3.051**	**0.003**
Status: V<Non	0.035 [-0.021, 0.090]	1.227	0.222
**Status: M<V**	**-0.086 [-0.142, -0.027]**	**-2.890**	**0.004**
Stim_Gen: m<f	0.003 [-0.036, 0.042]	0.161	0.873
Rater_Gen: m<f	0.010 [-0.032, 0.052]	0.466	0.642
S_Gen*R_Gen: m<f	-0.032 [-0.079, 0.016]	-1.307	0.191
**Random Part**			
σ^2	0.554		
τ_0^2	0.004_{Stim}		
τ_0^2	0.003_{Sub}		
ICC	0.013		
N	190_{Stim} / 80_{Sub}		
Observations	15200		
Conditional R^2 / Marginal R^2	0.015 / 0.002		

Table S16. LMM results predicting Interestingness-Boost through mean facial Interestingness, interacting with the Status of the depicted person (musician, visual artist, non-artist). To test H3b, the following model has been fitted:

Level 1: $\text{Interestingness-Boost}_{ij} = \beta_{0j} + \beta_{i0} + \beta_{1j}\,\text{MeanFacialInterestingness}_{ij} * \beta_{2j}\,\text{Status}_{ij} + \varepsilon_{ij}$

	Interestingness-Boost		
Predictors	*Estimates [95%-CI]*	*t*	*p*
**Fixed Effects**			
(Intercept)	7.321 [5.684, 8.958]	8.752	<0.001
**MeanFacialInterest**	**-0.16920 [-0.286, -0.053]**	**0.060**	**0.005**
Status: M<Non	1.380 [-0.099, 2.860]	1.812	0.071
**Status: V>Non**	**7.177 [4.267, 10.087]**	**4.791**	**<0.001**
**Status: M<V**	**-5.796 [-8.789, -2.804]**	**-3.763**	**<0.001**
MFI*S: M<Non	-0.046 [-0.220, 0.128]	-0.517	0.605
MFI*S: V<Non	-0.154 [-0.530, 0.222]	-0.795	0.427
MFI*S: M<V	0.108 [-0.273, 0.488]	0.550	0.583
**Random Part**			
σ^2	436.76		
τ_0^2	18.05_{Stim}		
τ_0^2	37.93_{Sub}		
ICC	0.114		
N	190_{Stim} / 80_{Sub}		
Observations	15200		
Conditional R^2 / Marginal R^2	0.123 / 0.010		

Table S17. LMM results predicting Sympathy-Boost through mean facial Sympathy, interacting with the Status of the depicted person (musician, visual artist, non-artist). To test H3b, the following model has been fitted:

Level 1: $\text{Sympathy-Boost}_{ij} = \beta_{0j} + \beta_{i0} + \beta_{1j}\,\text{MeanFacialSympathy}_{ij} * \beta_{2j}\,\text{Status}_{ij} + \varepsilon_{ij}$

	Sympathy-Boost		
Predictors	*Estimates [95%-CI]*	*t*	*p*
**Fixed Effects**			
(Intercept)	4.994 [3.876, 6.113]	8.689	<0.001
MeanFacialSympathy	-0.026 [-0.101, 0.049]	-0.669	0.504
Status: M<Non	-0.348 [-1.864, 1.169]	-0.445	0.657
Status: V<Non	0.805 [-2.178, 3.789]	0.524	0.601
Status: M<V	-1.153 [-4.226, 1.921]	-0.728	0.468
MFS*S: M<Non	-0.096 [-0.228, 0.036]	-1.412	0.160
MFS*S: V<Non	-0.064 [-0.306, 0.179]	-0.508	0.612
MFS*S: M<V	-0.032 [-0.288, 0.223]	-0.247	0.805
**Random Part**			
σ^2	301.236		
$\tau_0{}^2$	13.562_{Stim}		
$\tau_0{}^2$	7.817_{Sub}		
ICC	0.066		
N	190_{Stim} / 80_{Sub}		
Observations	15200		
Conditional R^2 / Marginal R^2	0.068 / 0.002		

Table S18. LMM results predicting Trustworthiness-Boost through mean facial Trustworthiness, interacting with the Status of the depicted person (musician, visual artist, non-artist). To test H3b, the following model has been fitted:

Level 1: $\text{Trustworthiness-Boost}_{ij} = \beta_{0j} + \beta_{i0} + \beta_{1j}\,\text{MeanFacialTrustworthiness}_{ij} * \beta_{2j}\,\text{Status}_{ij} + \varepsilon_{ij}$

	Trustworthiness-Boost		
Predictors	*Estimates [95%-CI]*	*t*	*p*
**Fixed Effects**			
(Intercept)	7.12823 [5.832, 8.424]	10.708	<0.001
**MeanFacialTrust**	**-0.152 [-0.249, -0.056]**	**-3.070**	**0.002**
**Status: M<Non**	**-2.342 [-3.973, -0.711]**	**-2.788**	**0.006**
Status: V<Non	-0.945 [-4.434, 2.543]	-0.526	0.599
Status: M<V	-1.39631 [-4.964, 2.171]	-0.760	0.448
MFT*S: M<Non	-0.025 [-0.190, 0.140]	-0.290	0.772
MFT*S: V<Non	-0.045 [-0.353, 0.263]	-0.283	0.777
MFT*S: M<V	0.020 [-0.302, 0.342]	0.122	0.903
**Random Part**			
σ^2	323.38		
τ_0^2	13.84_{Stim}		
τ_0^2	13.17_{Sub}		
ICC	0.077		
N	190_{Stim} / 80_{Sub}		
Observations	15200		
Conditional R^2 / Marginal R^2	0.085 / 0.008		

Table S19. LMM results predicting Wish-to-Meet-Boost through mean facial value for Wish-to-Meet, interacting with the Status of the depicted person (musician, visual artist, non-artist). To test H3b, the following model has been fitted:

Level 1: $\text{Wish-to-Meet-Boost}_{ij} = \beta_{0j} + \beta_{i0} + \beta_{1j}\,\text{MeanFacialWish-to-Meet}_{ij} * \beta_{2j}\,\text{Status}_{ij} + \varepsilon_{ij}$

	Wish-to-Meet-Boost		
Predictors	*Estimates [95%-CI]*	*t*	*p*
**Fixed Effects**			
(Intercept)	5.06844 [3.962, 6.175]	8.945	<0.001
MeanFacialMeet	-0.085 [-0.169, -0.001]	-1.967	0.051
Status: M<Non	-0.293 [-1.446, 0.860]	-0.493	0.622
**Status: V>Non**	**3.357 [1.314, 5.401]**	**3.191**	**0.002**
**Status: M<V**	**-3.650 [-5.756, -1.545]**	**-3.368**	**<0.001**
MFM*S: M<Non	-0.084 [-0.217, 0.050]	-1.216	0.226
MFM*S: V<Non	-0.119 [-0.390, 0.152]	-0.854	0.394
MFM*S: M<V	0.036 [-0.242, 0.313]	0.249	0.804
**Random Part**			
σ^2	354.291		
τ_0^2	9.572_{Stim}		
τ_0^2	14.309_{Sub}		
ICC	0.063		
N	190_{Stim} / 80_{Sub}		
Observations	15200		
Conditional R^2 / Marginal R^2	0.068 / 0.005		

Table S20. LMM results predicting ViewingTime-Boost through mean facial ViewingTime, interacting with the Status of the depicted person (musician, visual artist, non-artist). To test H3b, the following model has been fitted:

Level 1: $\text{ViewingTime-Boost}_{ij} = \beta_{0j} + \beta_{i0} + \beta_{1j}\,\text{MeanFacialViewingTime}_{ij} * \beta_{2j}\,\text{Status}_{ij} + \varepsilon_{ij}$

	ViewingTime-Boost		
Predictors	*Estimates [95%-CI]*	*t*	*p*
**Fixed Effects**			
(Intercept)	6.888 [4.535, 9.241]	5.682	<0.001
**MeanFacialView**	**-0.687 [-0.925, -0.448]**	**-5.586**	**<0.001**
Status: M<Non	-1.029 [-5.058, 2.999]	-0.496	0.621
Status: V<Non	2.399 [-7.242, 12.040]	0.483	0.630
Status: M<V	-3.429 [-13.333, 6.476]	-0.672	0.503
MFV*S: M<Non	0.100 [-0.308, 0.508]	0.476	0.635
MFV*S: V<Non	-0.240 [-1.217, 0.738]	-0.476	0.635
MFV*S: M<V	0.340 [-0.664, 1.344]	0.656	0.512
**Random Part**			
σ^2	0.554		
$\tau_0{}^2$	0.002_{Stim}		
$\tau_0{}^2$	0.003_{Sub}		
ICC	0.009		
N	190_{Stim} / 80_{Sub}		
Observations	15200		
Conditional R^2 / Marginal R^2	0.014 / 0.005		

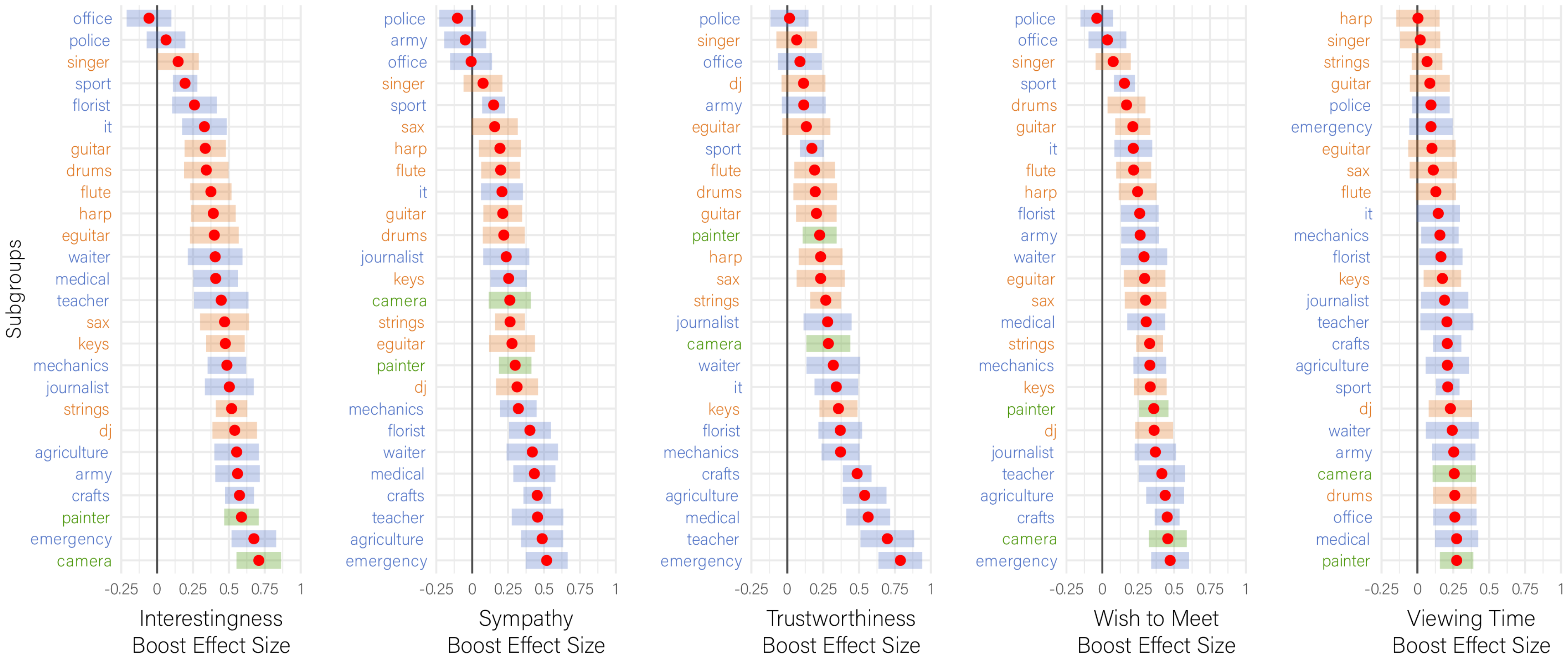

Figure S8. Effect sizes of boosts per subgroup for all remaining variables beside Attractiveness (which is shown in Fig. 6). The forest plots depict EMMs for the boosts expressed as Cohen's d for each stimulus and predicted in a linear model by subgroup membership. The error bars represent 95 % confidence limits. For better readability, the superordinate status membership of the subgroups is coded in color: non-artists are shown in blue, musicians in orange and visual artists in green.

Table S21. LMM results for the comprehensive covariates model (model.9 in R script) predicting Attractiveness through Layout and Status, while controlling for stimulus order, character's smile intensity, character's gaze direction, character's estimated income, character's estimated age, character's gender, rater's gender, rater's preference for professions and their own music production (today and overall, including their past).

	Attractiveness		
Predictors	*Estimates [95%-CI]*	*t*	*p*
**Fixed Effects**			
(Intercept)	17.239 [9.957, 24.518]	4.563	<0.001
**Layout**	**3.332 [2.872, 3.792]**	**14.201**	**<0.001**
Status: M<Non	-1.244 [-4.541, 2.052]	-0.730	0.466
Status: V<Non	-0.028 [-5.307, 5.249]	-0.010	0.991
Status: M<V	-1.266 [-3.117, 0.585]	-1.327	0.185
**Stimulus_Order**	**-0.019 [-0.022, -0.016]**	**-12.054**	**<0.001**
Income	-0.000 [-0.001, 0.000]	-0.642	0.521
**Prof_Pref**	**0.056 [0.047, 0.065]**	**12.532**	**<0.001**
Smile	1.037 [-0.111, 2.186]	1.745	0.082
**Gaze**	**-3.107 [-6.144, -0.070]**	**-1.976**	**0.049**
**Age**	**-0.402 [-0.581, -0.221]**	**-4.317**	**<0.001**
**Stim_Gender: M<F**	**-10.198 [-12.799, -7.598]**	**-7.575**	**<0.001**
Gender: M<F	-3.465 [-0.396, 7.327]	-1.738	0.086
**L*S: M<Non**	**-1.256 [-1.986, -0.526]**	**-3.372**	**<0.001**
L*S: V<Non	0.131 [-1.133, 1.395]	0.203	0.838
L*S: M<V	0.003 [-0.7, 0.706]	0.011	0.991
Status N : Leisure	1.373 [-0.737, 3.484]	1.261	0.211
Status M : Leisure	0.834 [-1.284, 2.953]	0.763	0.447
Status V : Leisure	0.859 [-1.331, 3.050]	0.760	0.449
Status N : Instrument	-0.546 [-1.969, 0.876]	-0.745	0.458
Status M : Instrument	-0.338 [-1.766, 1.089]	-0.459	0.647
Status V : Instrument	-0.114 [-1.591, 1.363]	-0.150	0.881

Table 21 [continued]

**Random Part**	
σ^2	231.24
τ_{0}^{2}	80.24_{Stim}
τ_{0}^{2}	77.39_{Sub}
ICC	0.405
N	190_{Stim} / 80_{Sub}
Observations	30400
Conditional R^2 / Marginal R^2	0.480 / 0.125

Table S22. Model comparison. The initial model (model.0) predicts the Attractiveness by the factors Layout and Status. It is Model-A as described in the manuscript. The consecutive models (model.1 to 9) append cumulatively one of the following covariates: stimulus order, estimated income of the depicted character, rater's preference for professions (Prof_Pref), character's smile intensity, character's gaze direction, character's estimated age, character's gender, rater's gender, rater's own musicality. The statistical model fit to the data is tested via the *anova* function in R.

			Statistical Model Fit			
Model	*n of parameters*	*added covariate*	*AIC*	*BIC*	*Chi squared*	*p*
model.0	9	[Layout * Status]	253297	253372		
model.1	10	Stim_Order	**253152**	**253235**	**147.081**	**<0.001**
**model.2**	**11**	**Income**	**253149**	**253241**	**5.022**	**0.025**
**model.3**	**12**	**Prof_Pref**	**253000**	**253100**	**151.225**	**<0.001**
**model.4**	**13**	**Smile**	**252998**	**253106**	**4.188**	**0.040**
model.5	14	Gaze	252997	253114	2.589	0.107
**model.6**	**15**	**Age**	**252979**	**253103**	**20.467**	**<0.001**
**model.7**	**16**	**Sim_Gender**	**252929**	**253062**	**51.871**	**<0.001**
model.8	17	Rater_Gender	252928	253069	2.792	0.094
model.9	23	Rater_Musicality	252930	253121	10.283	0.113

* AIC: Akaike information criterion, BIC: Bayesian information criterion
